# Epidemiology and Genomic Characterization of Two Novel SARS-Related Coronaviruses in Horseshoe Bats from Guangdong, China

**DOI:** 10.1128/mbio.00463-22

**Published:** 2022-04-25

**Authors:** Linmiao Li, Libiao Zhang, Jiabin Zhou, Xiangyang He, Yepin Yu, Ping Liu, Wenzhong Huang, Zuofu Xiang, Jinping Chen

**Affiliations:** a College of Life Science and Technology, Central South University of Forestry and Technology, Changsha, Hunan, China; b Guangdong Key Laboratory of Animal Conservation and Resource Utilization, Institute of Zoology, Guangdong Academy of Sciences, Guangzhou, Guangdong, China; c College of Forestry, Central South University of Forestry and Technology, Changsha, Hunan, China; College of Veterinary Medicine, Cornell University

**Keywords:** SARS-related coronavirus, epidemiology, genetic diversity, horseshoe bats

## Abstract

Severe acute respiratory syndrome (SARS) coronavirus (SARS-CoV) and SARS-CoV-2, the causative agents of SARS, which broke out in 2003, and coronavirus disease 2019 (COVID-2019), which broke out in 2019, probably originated in Rhinolophus sinicus and R. affinis, respectively. *Rhinolophus* bats are important hosts for coronaviruses. Many SARS-related coronaviruses (SARSr-CoVs) have been detected in bats from different areas of China; however, the diversity of bat SARSr-CoVs is increasing, and their transmission mechanisms have attracted much attention. Here, we report the findings of SARSr-CoVs in R. sinicus and *R. affinis* from South China from 2008 to 2021. The full-length genome sequences of the two novel SARSr-CoVs obtained from Guangdong shared 83 to 88% and 71 to 72% nucleotide identities with human SARS-CoV and SARS-CoV-2, respectively, while sharing high similarity with human SARS-CoV in hypervariable open reading frame 8 (ORF8). Significant recombination occurred between the two novel SARSr-CoVs. Phylogenetic analysis showed that the two novel bat SARSr-CoVs from Guangdong were more distant than the bat SARSr-CoVs from Yunnan to human SARS-CoV. We found that transmission in bats contributes more to virus diversity than time. Although our results of the sequence analysis of the receptor-binding motif (RBM) and the expression pattern of angiotensin-converting enzyme 2 (ACE2) inferred that these viruses could not directly infect humans, risks still exist after some unpredictable mutations. Thus, this study increased our understanding of the genetic diversity and transmission of SARSr-CoVs carried by bats in the field.

## INTRODUCTION

Emerging infectious diseases (EIDs) pose a great threat to global public health. Approximately 60% to 80% of human EIDs originate from wildlife, and bats may be natural reservoirs for a large variety of viruses, including many important zoonotic viruses that cause severe diseases in humans and domestic animals, especially severe acute respiratory syndrome (SARS) coronavirus (SARS-CoV), Middle East respiratory syndrome coronavirus (MERS-CoV), and SARS-CoV-2, which caused outbreaks in various countries (1–4). These three coronaviruses spread across species through one or more intermediate hosts to achieve zoonotic spillover, during which viral mutation, recombination, and/or amplification may occur (5–7). It is worth noting that horseshoe bats (Rhinolophidae) are thought to be the natural hosts of SARS-CoV and SARS-CoV-2. Some research also demonstrated that *Rhinolophus* bats may be important hosts for coronaviruses (8, 9).

Coronavirus diversity in bats is thought to be shaped by both species richness and geographic distribution. In China, horseshoe bat species are widely distributed and are also the most frequent SARS-related coronavirus (SARSr-CoV) carriers (7, 10–12), particularly Rhinolophus sinicus and R. affinis. Bats in Yunnan have been found to carry the most SARSr-CoVs, some of which have been successfully isolated and proven to use human angiotensin-converting enzyme 2 (hACE2) as the receptor for viral internalization (13–15). Through whole-genome sequence analysis, the origin and evolution of the virus can be better traced. Besides, the complete genomic sequences of SARSr-CoVs have been obtained from *Rhinolophus* bats in some provinces, so the full-length genome sequence of the SARSr-CoV strain carried by bats from Guangdong is urgently needed for virus traceability.

South China, especially Guangdong province, is rich in wildlife. There are more than 60 species of bats in Guangdong. Although SARS-CoV was first reported in humans in Guangdong and caused fatal respiratory infections in close to 800 people worldwide in 2002, subsequent investigations have identified horseshoe bats (genus *Rhinolophus*) from Yunnan as the natural reservoirs of SARS-CoV (13). In 2016, swine acute diarrhea syndrome coronavirus (SADS-CoV) caused the death of over 25,000 pigs in farms within Guangdong province. This virus originated from *Rhinolophus* species bats (16). As a natural reservoir of viruses, bats harbor numerous other SARSr-CoVs that could potentially infect humans around the world, causing a SARS- or coronavirus disease 2019 (COVID-19)-like pandemic in the future. Thus, it is necessary to discover more virus diversity to avoid future outbreaks. South China was also a hub for illegal wildlife trade; therefore, it is very necessary to investigate and understand the diversity and transmission of coronaviruses carried by *Rhinolophus* species bats from South China to prevent a public health event.

## RESULTS

### Epidemiology of SARSr-CoVs in *Rhinolophus* bats from South China.

A total of 389 anal swabs from 201 *R. sinicus*, 177 *R. affinis*, and 11 Hipposideros pomona bats were collected in roost caves in Guangdong (including Guangzhou, Huizhou, and Shenzhen) and Hainan (see Table S1 in the supplemental material), over the 12-year study period. These specimens were tested for the presence of betacoronaviruses by Reverse Transcription-Polymerase Chain Reaction (RT-PCR) targeting the 360-nucleotide (nt) S gene fragment. In total, 22 samples from *R. sinicus* (17 individuals from Guangzhou and 5 individuals from Huizhou near Guangzhou) and 6 samples from *R. affinis* (5 individuals from Guangzhou and 1 from Huizhou) tested positive for betacoronaviruses (Table 1; Table S1). Sequencing of the PCR amplicons revealed that the 28 sequences were bat SARSr-CoVs closely related to strains from Guangdong, Jiangxi, Fujian, and Hongkong (Fig. S1).

**TABLE 1:** Results of samples for CoV testing by RT-PCR

Location^*a*^	Date (mo and yr)	Rhinolophus sinicus			Rhinolophus affinis			Hipposideros pomona		
		No. of samples	No. of betacoronavirus-positive samples	Positivity rate (%)	No. of samples	No. of betacoronavirus-positive samples	Positivity rate (%)	No. of samples	No. of betacoronavirus-positive samples	Positivity rate (%)
Guangzhou	November 2009	11	2	18.18	0	0	0	0	0	0
	April 2020	41	5	12.20	15	0	0	0	0	0
	September 2020	34	5	14.71	12	3	0.25	0	0	0
	December 2020	0	0	0.00	0	0	0	8	0	0
	March 2021	31	5	16.13	41	2	4.88	3	0	0
	May 2021	3	0	0.00	17	0	0	0	0	0

Huizhou	October 2012	2	0	0.00	0	0	0	0	0	0
	May and June 2013	28	2	7.14	18	1	5.56	0	0	0
	May and June 2020	3	2	66.67	61	0	0	0	0	0
	August 2020	4	1	25.00	0	0	0	0	0	0

Shenzhen	September 2020	15	0	0.00	13	0	0	0	0	0

Hainan	July 2008	29	0	0.00	0	0	0	0	0	0


Total		201	22	10.95	177	6	3.39	11	0	0

a Guangzhou, Huizhou, and Shenzhen are the cities in Guangdong.

10.1128/mbio.00463-22.1 FIG S1 Phylogenetic tree based on nucleotide sequences obtained by RT-PCR using ZH2 primers. The tree was constructed by the maximum likelihood method using the MrBayes approach employing the GTR+I+G nucleotide substitution model. The red letters represent the SARSr-CoV strains isolated from Rhinolophus sinicus; the blue letters represent the SARSr-CoV strains isolated from *R. affinis*. Download FIG S1, PDF file, 0.2 MB. Copyright © 2022 Li et al. 2022 Li et al. https://creativecommons.org/licenses/by/4.0/ This content is distributed under the terms of the Creative Commons Attribution 4.0 International license.

10.1128/mbio.00463-22.7 TABLE S1 Sample information of bats. Download Table S1, PDF file, 0.4 MB. Copyright © 2022 Li et al. 2022 Li et al. https://creativecommons.org/licenses/by/4.0/ This content is distributed under the terms of the Creative Commons Attribution 4.0 International license.

To understand the genetic diversity of these bat SARSr-CoVs, the complete S genes of the SARSr-CoV strains were amplified and sequenced. Due to the low viral loads in some samples, S gene sequences were successfully amplified from 7 samples, including Rs56, Rs67, Rs68, Rs87, RaCH025, RaCH027, and RaCH039 from Guangzhou, and most of the S gene sequences were obtained from 1 sample (Rs200609) from Huizhou in 2020 and 2 samples (Rs150 and Rs183) from Guangzhou in 2009. These S gene sequences showed high similarity among themselves. Interestingly, compared with positive samples (Rs56, Rs67, Rs68, Rs87, RaCH025, RaCH027, and RaCH039) from Guangzhou in 2020, the virus sequences of samples (Rs150 and Rs183) from Guangzhou in 2009 were similar, suggesting that this virus may have existed for a long period and is almost conserved in the Guangzhou population (Fig. S2). However, different from the S gene sequence of SARSr-CoV isolated from bats from Huizhou, we found 17 amino acid (aa) variation sites, including a 1-aa insertion site in the Rs200609 sequence compared with the sequences of Rs56/67/68/87 (Fig. S3), which clearly suggested that SARSr-CoV may be mutated by transmission from different geographical populations. But in the same geographical location, the SARSr-CoV carried by *R. sinicus* (Rs56/87/67/68) and *R. affinis* (RaCH025/27/39) also has a 1-aa variation (Fig. S3), suggesting that host switching increases the mutation of the virus.

10.1128/mbio.00463-22.2 FIG S2 Sequence alignment of SARSr-CoVs from Guangzhou in 2009 and 2020. Download FIG S2, PDF file, 0.3 MB. Copyright © 2022 Li et al. 2022 Li et al. https://creativecommons.org/licenses/by/4.0/ This content is distributed under the terms of the Creative Commons Attribution 4.0 International license.

10.1128/mbio.00463-22.3 FIG S3 Amino acid sequence comparison of S genes of SARSr-CoVs from Guangzhou and Huizhou, Guangdong province. Download FIG S3, PDF file, 0.6 MB. Copyright © 2022 Li et al. 2022 Li et al. https://creativecommons.org/licenses/by/4.0/ This content is distributed under the terms of the Creative Commons Attribution 4.0 International license.

### Viral metagenomics.

Viral nucleic acids of Rs56 were deep sequenced, and we then obtained a total of 11.6 Gb of data (13,571,072 clean reads; 150 bp in length). In total, 1,155,340 reads were best matched with viral proteins available in the NCBI NR database. The most widely distributed virus family was *Coronaviridae*, and the diverse reads related to this family occupied ∼97.92% of the total viral sequence reads (Fig. S4). Contig sequences were then generated by *de novo* assembly using MEGAHIT version 1.0 (17), generating 10,588 unique contigs. A taxonomic assignment of these contigs was performed based on BLAST analysis. At this stage, 6 contigs were confirmed for RNA virus species (Table 2), 1 of which was a novel SARSr-CoV with a length of 30,146 nt.

**TABLE 2:** Information on contigs with a high level of sequence similarity and then confirmed to be *Coronavirinae*^*a*^

Query ID	Subject ID (GenBank accession no.)	Identity (%)	Alignment length (nt)	No. of mismatches	No. of gap openings	q_start (position)	q_end (position)	s_start (position)	s_end (position)	E value	Bit score	Taxonomy
Contig_4536	GQ153542.1	93.33	27,752	1,845	4	231	27978	2	27751	0	41,690	SARSr-CoV
Contig_5449	DQ022305.2	98.333	240	4	0	122	361	29099	28860	9.60 × 10^−114^	416	SARSr-CoV
Contig_3133	KY770859.1	98.246	228	3	1	127	353	27732	27959	3.73 × 10^−106^	390	Bat CoV
Contig_6085	MK211378.1	98.578	211	2	1	522	731	30042	29832	1.13 × 10^−97^	364	Bat CoV/YN2018D
Contig_9629	MK211378.1	100	126	0	0	577	702	30163	30038	5.61 × 10^−57^	228	Bat CoV/YN2018D
Contig_9617	AY536760.3	94.048	168	6	2	3	167	184	18	7.78 × 10^−65^	253	SARSr-CoV

a Query ID: query sequence ID; Subject ID: the target sequence ID; Identity (%): the percentage of consistency of sequence alignment; Alignment length: the length of the matched area; No. of mismatches: the number of mismatch bases in the matched area; No. of gap openings: the number of gaps in the matched area; q_ start: the starting point of the comparison region on the query sequence; q_end: the termination point of the matched area on the query sequence; s_start: the starting position of the matching region on the subject sequence; s_end: the termination site of the matched area on the subject sequence; E value: the expected value of the comparison result; Bit score: the bit score value of the comparison result; Taxonomy: Annotation information.

10.1128/mbio.00463-22.4 FIG S4 Virus reads classified at the family level by viral metagenomics. Download FIG S4, PDF file, 0.1 MB. Copyright © 2022 Li et al. 2022 Li et al. https://creativecommons.org/licenses/by/4.0/ This content is distributed under the terms of the Creative Commons Attribution 4.0 International license.

### Genomic characterization of the novel SARSr-CoVs.

**FIG 1:**
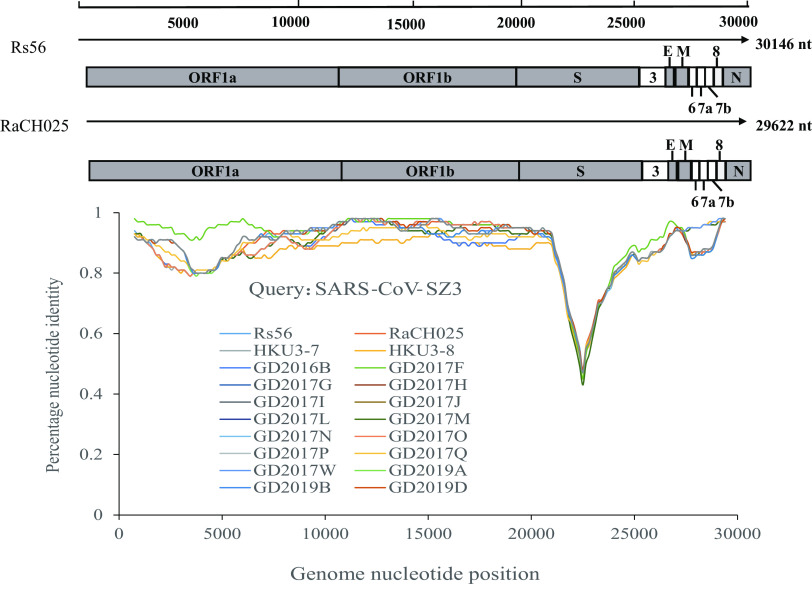
Gene map of the two novel SARSr-CoVs and similarity plot based on the full-length genome sequence of SARS-CoV-SZ3. Full-length genome sequences of Bat-SARSr-CoV-RaCH025, Bat-SARSr-CoV-Rs56, Bat-SARSr-CoV-HKU3-7/HKU3-8, and Bat-SARSr-CoV-GD2016B/2017F/2017G/2017H/2017I/2017J/2017L/2017M/2017N/2017O/2017P/2017Q/2017W/2019A/2019B/2019D were used as reference sequences. The analysis was performed with the Kimura model, with a window size of 1,500 bp and a step size of 150 bp.

The full-length genomes of two novel bat SARSr-CoVs, strains named by abbreviation of the bat species and sample identifier (RaCH025 and Rs56), were 29,622 nt and 30,146 nt, respectively, and the gene structure was similar to those of SARS-CoV and other bat SARSr-CoVs (Fig. 1). Rs56 was assembled by high-throughput sequencing (contig_4536) (Table 2) and verified to be 29,674 nt by RT-PCR with 70 pairs of feasible primers (Table S2A) without the 255 nt at the 5′ terminus and the 217 nt at the 3′ terminus. However, the complete genome of the RaCH025 strain was obtained by RT-PCR with 64 pairs of feasible primers (Table S2B). The genomes of the two SARSr-CoVs in the same cave shared 97.95% nucleotide sequence identity, and the main difference existed in open reading frame 8 (ORF8), with a similarity of only 34.43% (Table 3). The overall nucleotide sequence identities between the two novel SARSr-CoVs and human SARS-CoV and SARS-CoV-2 were 83 to 88% and 71 to 72%, respectively, lower than that observed for bat SARSr-CoVs reported from other locations in China (88 to 96%) (6, 7, 10, 12, 18, 19). Comparatively speaking, Rs56 and RaCH025 had high similarity with 18 strains downloaded from the NCBI database from Guangdong with complete sequences (Table S3), which was examined by Simplot analysis (Fig. 1).

**TABLE 3:** Nucleotide and protein sequence identities among the whole genome and each gene or region of SARS-CoV, SARS-CoV-2, and other bat SARSr-CoVs against Bat-SARSr-CoV-Rs56

Virus name	Nucleotide/amino acid sequence identity (%)^*a*^												
	Whole genome	S	RBD	E	M	N	ORF1ab	RdRp	ORF3a	ORF6	ORF7a	ORF7b	ORF8
Human-SARS-CoV-2	72.95/81.20	65.57/73.65	62.05/69.12	92.11/92	81.23/90.05	87.14/89.50	75.41/86.24	86.27/96.23	70.91/72.99	69.89/68.85	81.15/85.12	/	32.79/20.66
Human-SARS-CoV-GZ02	87.98/90.51	72.76/78.80	58.20/58.92	97.40/100	93.54/96.33	95.97/96.91	91.63/96.08	94.28/97.35	82.42/85.04	93.75/92.06	91.87/92.62	92.59/93.18	97.29/97.54
Civet-SARS-CoV-civet010	87.82/90.42	72.62/78.57	58.90/59.46	97.40/100	93.09/95.93	95.89/96.67	91.60/95.96	92.76/95.86	81.45/83.21	93.75/92.06	91.87/92.62	92.59/93.18	97.29/97.54
**Bat-SARSr-CoV-RaCH025/Guangdong**	97.95/98.44	99.73/99.92	99.84/100	99.57/100	97.30/99.09	98.34/99.29	98.62/99.28	99.89/99.78	99.88/99.64	96.88/98.41	98.37/99.18	100/100	34.43/25.62
Bat-SARSr-CoV-WIVI/Yunnan	82.22/88.90	73.08/79.37	59.61/61.08	97.84/100	94.14/98.19	95.89/96.67	92.14/96.24	92.94/95.86	82.30/85.04	92.19/90.48	92.14/95.90	91.85/95.45	31.14/24.79
Bat-SARSr-CoV-YN2013/Yunnan	90.62/94.72	78.20/85.32	80.62/90.20	97.83/100	92.19/95.02	96.29/97.15	92.23/96.41	94.96/97.73	86.06/89.78	94.79/88.00	91.87/95.08	89.63/93.18	98.37/98.36
Bat-SARSr-CoV-Rs6272006/Guizhou	90.39/91.20	83.60/91.70	77.78/86.49	97.83/100	93.84/97.74	96.60/97.39	92.00/96.09	92.97/95.97	86.79/89.78	93.75/93.65	93.77/96.72	91.85/95.45	31.14/24.79
Bat-SARSr-CoV-GX2013/Guangxi	93.53/96.83	91.92/97.26	80.24/88.11	98.70/100	94.89/97.28	96.52/97.39	93.30/96.73	92.94/95.86	94.19/95.26	94.79/92.06	96.21/98.36	96.29/100	98.10/96.72
Bat-SARSr-CoV-HuB2013/Hubei	88.92/73.22	80.19/88.72	74.60/85.41	98.70/98.68	95.80/97.74	96.28/97.38	90.16/97.03	88.56/95.44	90.06/94.16	97.40/100	90.51/95.90	91.11/95.45	33.06/22.31
Bat-SARSr-CoV-HKU3-1/Hongkong	92.52/96.84	94.65/97.82	82.01/88.11	100/100	96.85/97.74	97.47/98.10	92.17/97.76	90.29/96.60	96.97/95.99	99.48/98.41	95.66/97.54	98.52/100	34.15/24.79
Bat-SARSr-CoV-ZXC21/Zhejiang	76.82/86.78	72.81/97.82	69.66/82.16	91.67/92.00	80.18/90.05	88.73/90.93	78.97/86.75	90.75/96.60	70.91/71.53	65.57/69.35	77.87/80.99	71.21/74.41	35.25/20.66

a RdRp, RNA-dependent RNA polymerase. RBD, Receptor-Binding Domain. “/” is used to separate the data before and after. Bold in the column of virus name represents the strain obtained in this study.

10.1128/mbio.00463-22.8 TABLE S2 Primers used to obtain the complete sequence and S gene of bat SARSr-CoV. (A) Primers used to obtain the complete sequence of Bat-SARSr-CoV-Rs56. (B) Primers used to obtain the complete sequence of Bat-SARSr-CoV-RaCH025. (C) Primers used to obtain the S gene of CoVs from positive samples. Download Table S2, PDF file, 0.4 MB. Copyright © 2022 Li et al. 2022 Li et al. https://creativecommons.org/licenses/by/4.0/ This content is distributed under the terms of the Creative Commons Attribution 4.0 International license.

10.1128/mbio.00463-22.9 TABLE S3 Information on betacoronaviruses in the GenBank database. Download Table S3, PDF file, 0.2 MB. Copyright © 2022 Li et al. 2022 Li et al. https://creativecommons.org/licenses/by/4.0/ This content is distributed under the terms of the Creative Commons Attribution 4.0 International license.

### Evolution analysis of SARSr-CoVs.

**FIG 2:**
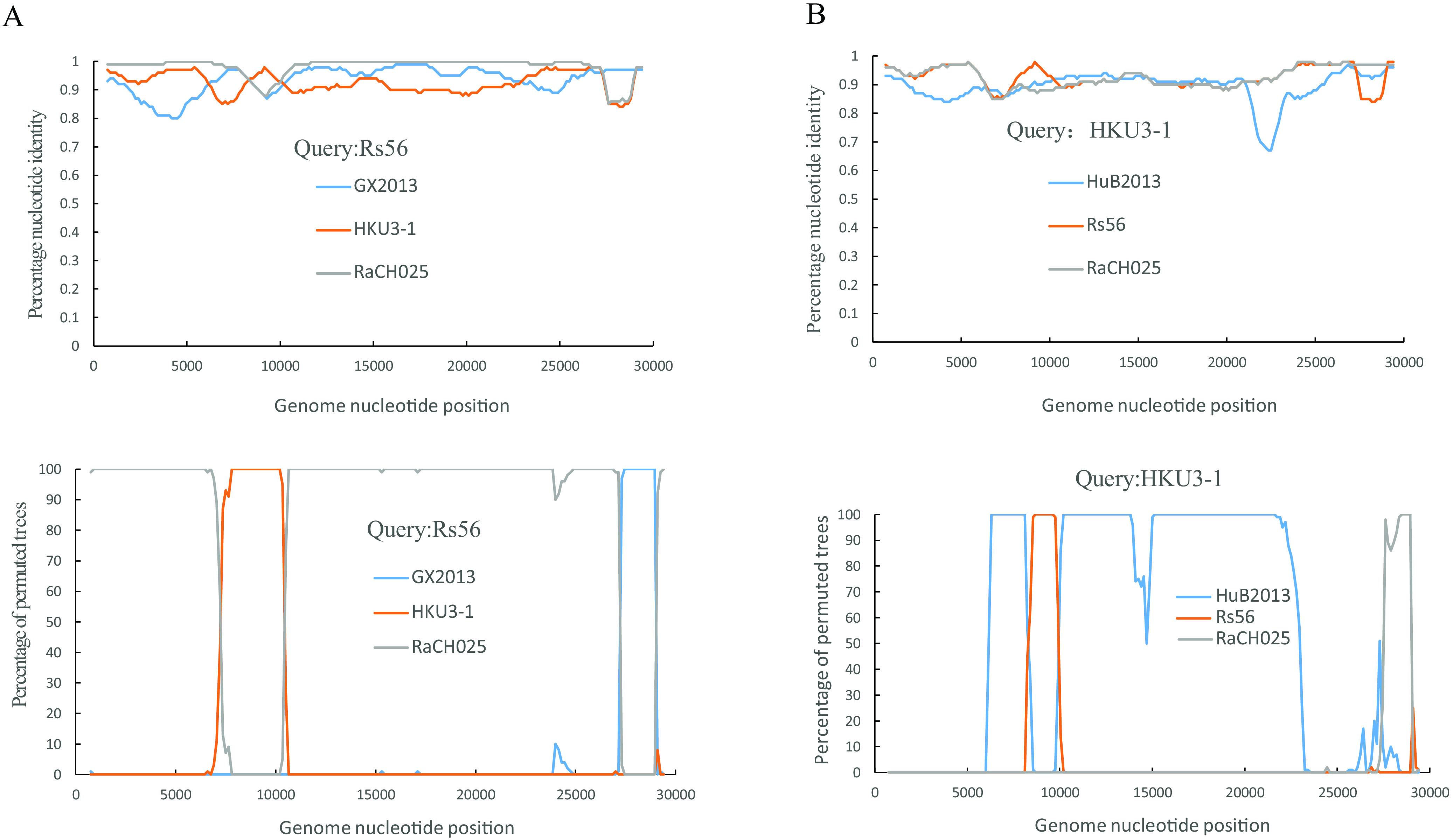
Detection of potential recombination events by similarity plot and bootscan analyses. (A) The full-length genome sequence of SARSr-CoV-Rs56 was used as the query sequence, and the sequences of GX2013, HKU3-1, and RaCH025 were used as the reference sequences. (B) The full-length genome sequence of HKU3-1 was used as the query sequence, and SARSr-CoVs HuB2013, Rs56, and RaCH025 were used as the reference sequences. All analyses were performed with a Kimura model, with a window size of 1,500 bp and a step size of 150 bp.

The potential recombination events between Rs56, RaCH025, HKU3-1, GX2013, and HuB2013 were examined by RDP4 and Simplot. Evidence of a recombination event was detected in the genome of the novel SARSr-CoV RaCH025; Rs56 and HKU3-1 were suggested to be the major and minor parents of RaCH025, respectively (*P* value of <10^−130^), and RaCH025 and HKU3-1 were suggested to be the major and minor parents of Rs56, with a strong *P* value (<10^−100^). The results suggested that significant recombination occurred between RaCH025 and Rs56 from bats in the same cave, and Rs56 was likely to be a recombinant strain from three SARSr-CoVs harbored by bats, namely, RaCH025, HKU3-1, and GX2013, with four breakpoints at genome positions 7350, 10500, 27300, and 29100 (Fig. 2A). The major and minor parents of GX2013 were RaCH025 and HKU3-1, with a strong *P* value (<10^−60^). However, HKU3-1 was likely to be a recombinant strain from two SARSr-CoVs harbored by bats, namely, Rs56 and HuB2013, with a strong *P* value (<10^−40^), and breakpoints were identified at genome positions 8400, 10050, and 27600 (Fig. 2B). The results also revealed that the SARSr-CoVs carried by bats in Guangdong had recombined and genetically mutated with those carried by bats in surrounding areas.

**FIG 3:**
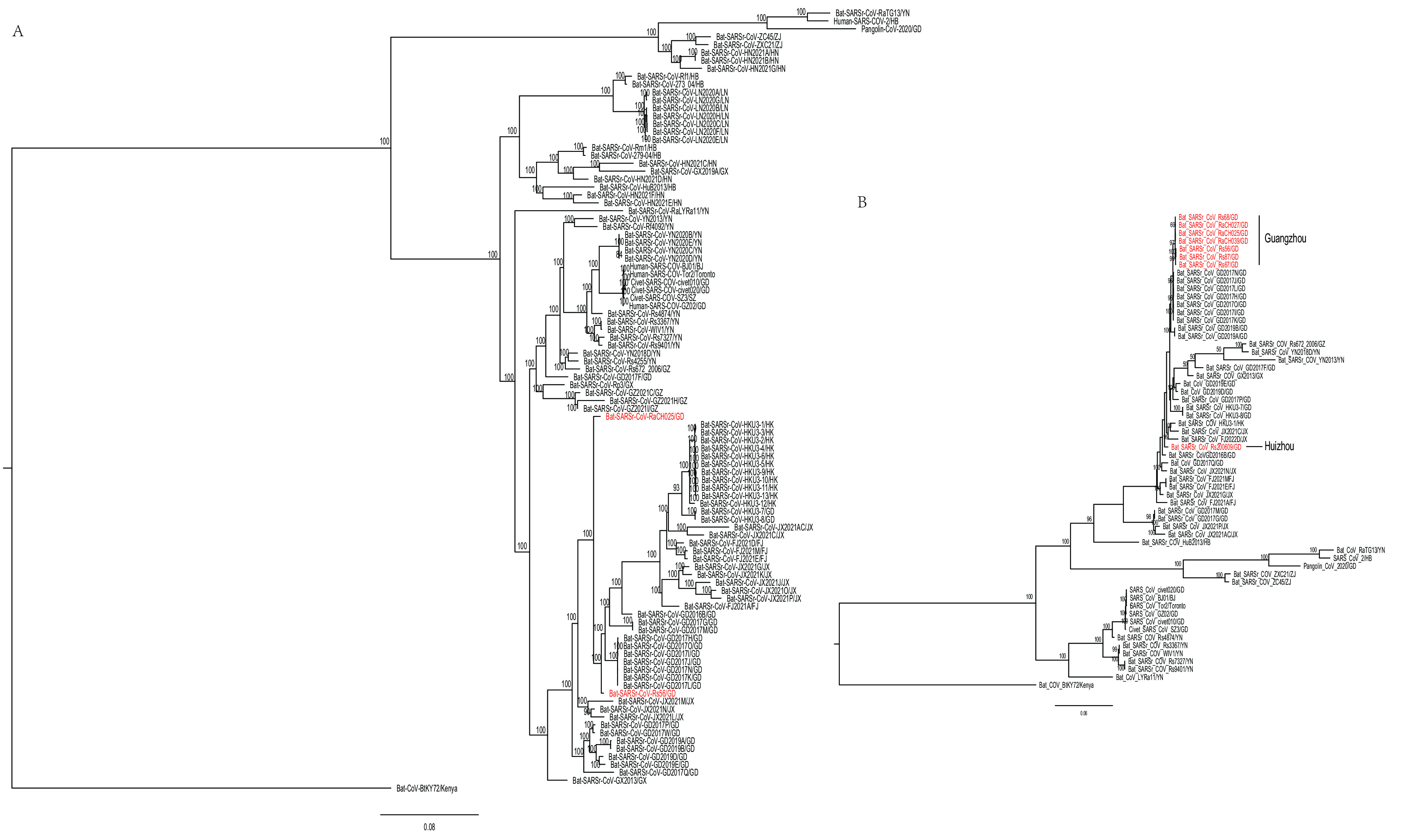
Phylogenetic trees based on nucleotide sequences of the whole genome (A) and the S gene (B). The trees were constructed by the maximum likelihood method using the MrBayes approach employing the GTR+I+G nucleotide substitution model. The red letters represent the SARSr-CoV strains isolated from Guangdong in this study. GD, Guangdong; FJ, Fujian; JX, Jiangxi; HK, Hongkong; HB, Hubei; YN, Yunnan; GX, Guangxi; GZ, Guizhou; LN, Liaoning; HN, Hunan; ZJ, Zhejiang.

Phylogenetic trees were constructed using the nucleotides of the complete genome, the ORF1ab gene, the S gene, and the ORF8 gene. Phylogenetic analysis based on the whole genomic sequence showed that the SARSr-CoVs carried by *Rhinolophus* species bats have genetic diversity and appear to be associated with host geographic distribution. The two novel SARSr-CoVs acquired in this study formed one clade with other strains from Hongkong, Guangdong, Jiangxi, Fujian, and Guangxi, which showed a more distant phylogenetic relationship to SARS-CoV than the bat SARSr-CoVs from Yunnan (Fig. 3A). The tree topology observed in the nonstructural protein gene ORF1ab was not completely consistent with the tree constructed by the whole-genome sequence, but two novel SARSr-CoVs acquired in this study were also more distant from SARS-CoV than the bat SARSr-CoVs from Yunnan (Fig. S5A). Based on the high genetic diversity of the S gene, nearly all bat SARSr-CoVs from Guangzhou were closely clustered; however, the bat SARSr-CoV from Huizhou was clustered into another branch, which showed that geographical differences exist in these CoVs in Guangdong (Fig. 3B). The result showed that the relationship of Rs56 formed in one clade with GX2013, GD2017M/G, and JX2021M/N, but that of RaCH025 formed in another clade in the ORF8 region (Fig. S5B). Especially, Rs56, GX2013, YN2013, GD2017M/G/W, and JX2021M/N/P/O/A/J/K from Chinese horseshoe bats were close to SARS-CoV according to the phylogenetic tree of ORF8, suggesting a critical role for Chinese horseshoe bats in the maintenance of SARS-CoVs.

10.1128/mbio.00463-22.5 FIG S5 Phylogenetic trees based on nucleotide sequences of ORF1ab (A) and ORF8 (B). The trees were constructed by the maximum likelihood method using the MrBayes approach employing the GTR+I+G (A) and GTR+G (B) nucleotide substitution models. The red letters represent the SARSr-CoV strains isolated from bats from Guangdong. Download FIG S5, PDF file, 0.2 MB. Copyright © 2022 Li et al. 2022 Li et al. https://creativecommons.org/licenses/by/4.0/ This content is distributed under the terms of the Creative Commons Attribution 4.0 International license.

### Cross-species transmission capacity of the bat SARSr-CoVs from Guangdong.

*R. sinicus* and *R. affinis* belong to the bat genus *Rhinolophus* and are regarded as the primary natural hosts of SARS-CoV and SARS-CoV-2, respectively. In the field survey, we found two interesting bat roost caves in Guangzhou. The relative positions of these two roost caves in the mountain were up and down, with about 5 m of vertical distance (Fig. S6). We conducted field surveys on the two caves from 2013 to 2021 and found that *R. sinicus* and *R. affinis* lived in the caves together except during hibernation periods, when they moved to other areas for hibernation in winter. However, in our study, *R. sinicus* and *R. affinis* bats in which SARSr-CoVs were detected did not display obvious signs of disease, but there was a 2.05% nucleotide sequence difference between the genomes of the two SARSr-CoVs from the same cave. Thus, the accumulation of variation could increase the risk of cross-species transmission.

10.1128/mbio.00463-22.6 FIG S6 Bat forms and schematic diagram of the bat roosting cave. (A) Rhinolophus sinicus and *R. affinis*. (B) Schematic diagram of the bat roosting cave. Download FIG S6, PDF file, 1.0 MB. Copyright © 2022 Li et al. 2022 Li et al. https://creativecommons.org/licenses/by/4.0/ This content is distributed under the terms of the Creative Commons Attribution 4.0 International license.

**FIG 4:**
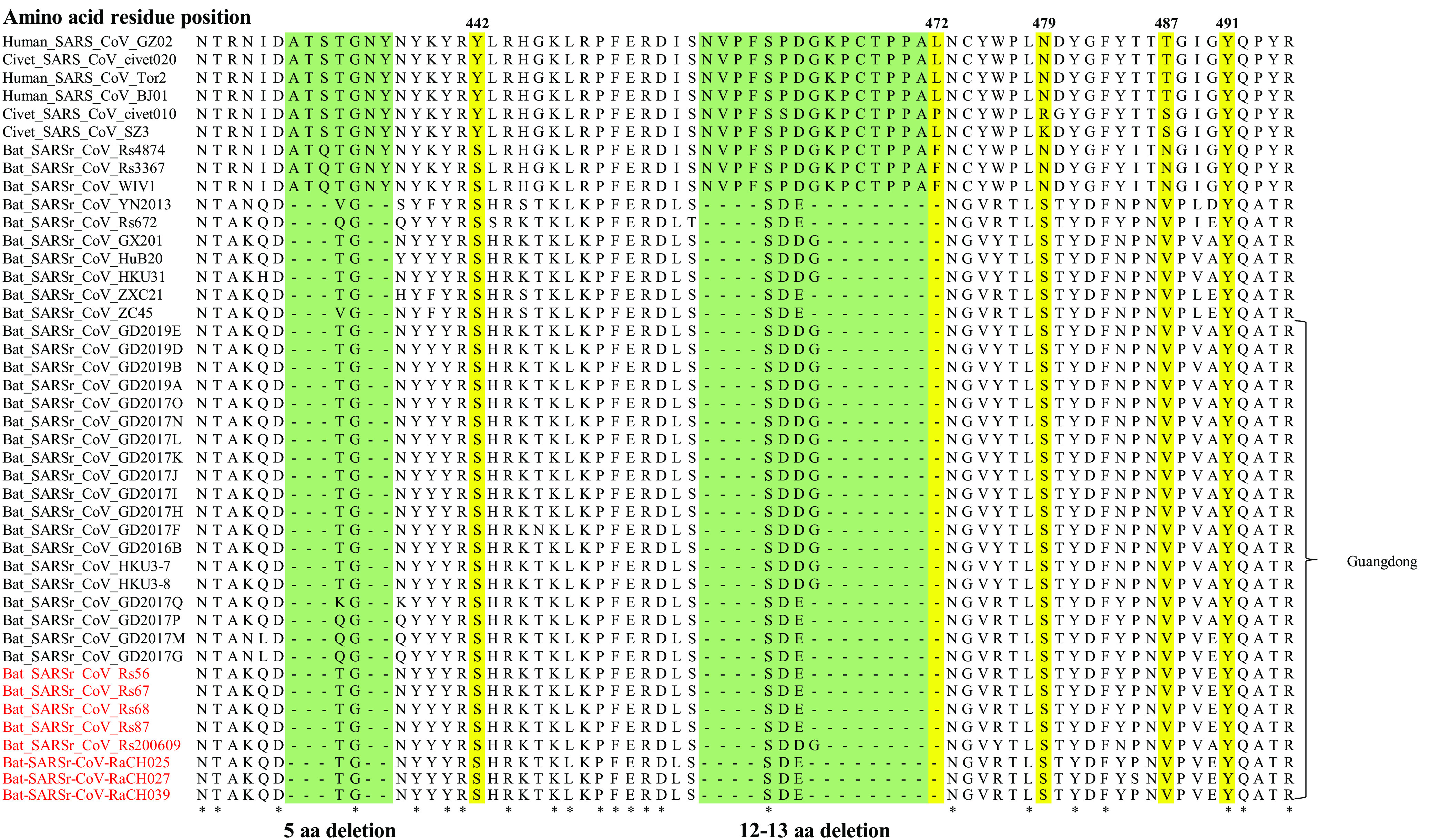
Sequence alignment of the receptor-binding motif (RBM) region. The red font represents the sequences obtained in this study. Yellow highlighting represents the 5 key sites where SARS-CoV binds to angiotensin-converting enzyme 2 (ACE2). Green shading represents amino acid (aa) deletions. The red words represent the sequences obtained in this study.

To date, reports confirm that the S protein is responsible for the entry of the virus and is functionally divided into two domains, S1 and S2, which are responsible for receptor binding and cellular membrane fusion, respectively. S1 consists of two domains, the N-terminal domain (NTD) and the C-terminal domain (CTD), which is also known as the receptor-binding domain (RBD) in SARS-CoV. The S proteins of SARSr-CoV-Rs56 and -RaCH025 showed 78.80–78.88% and 78.57–78.64% amino sequence identities to those of human and civet SARS-CoVs, and their RBD proteins were 58.92% and 59.46% similar to those of human and civet SARS-CoVs, respectively. In the receptor-binding motif (RBM) region, among the five key binding sites (residues 442, 472, 479, 487, and 491) with the receptor ACE2 (20), only residue 491 was conserved (Fig. 4). Bat-SARSr-CoV-Rs4874/Rs3367/WIVI strains from Yunnan have been verified by experiments to be able to use hACE2 as the entry receptor. Compared to them, bat SARSr-CoV strains from Guangdong failed to bind hACE2 because of the presence of two deletions (5 aa and 12-13 aa) in the key regions of RBMs, which was consistent with other bat SARSr-CoVs such as SARSr-CoV-YN2013, SARSr-CoV-Rs672, SARSr-CoV-GX2013, SARSr-CoV-HuB2013, SARSr-CoV-HKU3-1, SARSr-CoV-ZXC21, and SARSr-CoV-ZC45 (Fig. 4). Thus, the SARSr-CoVs carried by bats in Guangdong could spread within their own population, but they cannot directly infect humans.

**FIG 5:**
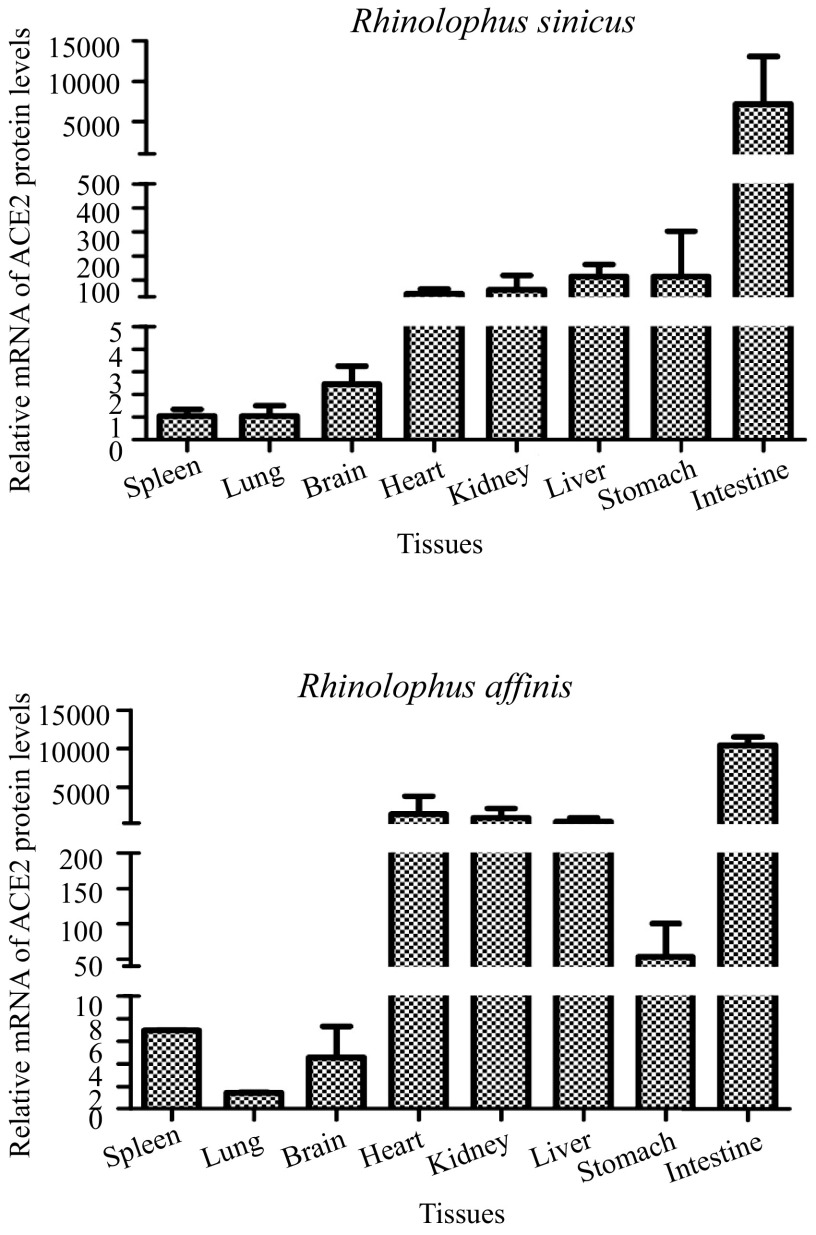
mRNA expression of ACE2 in different organs of Rhinolophus sinicus and *R. affinis*.

ACE2 had great genetic diversity in bats, and their receptor usage is species dependent, so the pathogenicity and host range of bat SARSr-CoVs remain to be determined in the future. The previous research of the interaction of the S protein between ACE2 and SARS-CoV/SARS-CoV-2 indicated that 23/22 sites on ACE2 can bind to the S protein receptor-binding region (21). We compared and analyzed the 24 amino acid sites, and the results showed that *R. sinicus* and *R. affinis* contain 6 or 8 sites with different amino acid residues compared to human ACE2 (Table 4), so we inferred that humans are susceptible to bat SARSr-CoVs. In this study, we also investigated the mRNA expression levels of ACE2 in eight organs of *R. sinicus* and *R. affinis* using quantitative real-time-PCR (qRT-PCR) and found that the mRNA expression levels of ACE2 were highest in the intestine; moreover, its expression levels were also high in the heart, kidney, and liver (Fig. 5). Among these organs, *R. sinicus* and *R. affinis* exhibited the lowest mRNA levels in the lung, followed by the spleen and brain. Our results suggested that all these organs are at risk of SARSr-CoV infection and that the intestine was the main channel for the spread of the virus.

**TABLE 4:**
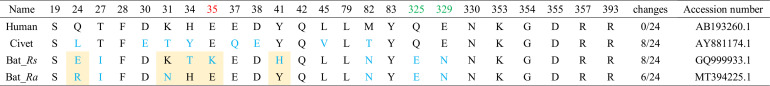
Sequence comparison among different ACE2s at the critical amino acid site that binds the SARS-CoV and SARS-CoV-2 receptor-binding domains^*a*^

a Note that black numbers indicate contact with SARS-CoV-2 S and SARS-CoV S, red numbers indicate contact with SARS-CoV-2 S only, and green numbers indicate contact with SARS-CoV S only (21). Amino acid differences compared to hACE2 are indicated as blue characters. The yellow shading represents the different sites between *R. sinicus* and *R. affinis*. *Rs*, *R. sinicus*; *Ra*, *R. affinis*.

## DISCUSSION

Here, we discovered two novel bat SARSr-CoVs, which were 2% to 7% different from those previously reported in Guangdong province (22). Through evolutionary and recombination analyses, these viruses from Guangdong might be recombined by viruses carried by bats from surrounding areas such as Hongkong, Guangxi and Hubei provinces. Guangdong and Guangxi are important hubs for the spread of coronavirus. The outbreak of SARS in Guangdong caused a severe pandemic since 2002. Evidence revealed that palm civets (Paguma larvata) may be the intermediate host. Although *R. sinicus* in Yunnan has been found to be the natural host of SARS, the viruses carried by bats can spread across species and mutate quickly. In addition, the illegal wildlife trade flourishes in Guangdong. Therefore, the long-term monitoring of the coronaviruses and even other viruses carried by bats in Guangdong is very important.

In our study, the region with the largest difference between the sequences of the two novel SARSr-CoVs found in bats from Guangdong was ORF8, and evolutionary analysis of ORF8 suggested that the two viruses are located in two different clades. Previous studies have shown that ORF8 is the most hypervariable and could be divided into 3 types (type I ORF8, type II ORF8, and type III ORF8) in bat lineage B betacoronaviruses (23). According to currently available data, *R. sinicus* is the only bat species with SARSr-CoVs containing 2 different types of ORF8s (types I and III) and also the only bat species that harbors SARSr-CoVs with type I ORF8 rarely (23). In our study, ORF8 of 12 bat SARSr-CoV strains (including SARSr-CoV-Rs56) carried by *R. sinicus* from Guangdong, Jiangxi, and Fujian belonged to type I (see Fig. S5B in the supplemental material). They were the most nearly identical to those of 2 CoVs, SARSr-CoV-GX2013 and SARSr-CoV-YN2013. The results suggested that *R. sinicus* plays an important role in the maintenance of SARS-CoVs. The ORF8 region, unique to SARSr-CoVs, is prone to mutations or deletions during interspecies transmission (1, 24). Compared to ORF8 of bat SARSr-CoVs, ORF8 from human SARS-CoV is under strong positive selection during animal-to-human transmission (19). Gradual deletions in the ORF8 region of human SARS-CoVs found in the early phase, the middle phase, and the late phase of the epidemic of SARS did not apparently affect the survival of the virus (1, 24, 25). Thus, these viruses should be taken seriously and monitored to prevent the occurrence of an epidemic in the future.

Many previous studies demonstrated the capacity of bat SARSr-CoVs (such as WIVI and Rs4874) to use ACE2 orthologs for cell internalization and efficient replication in human cells, which indicates that diverse variants of SARSr-CoV S proteins without deletions in their RBDs are able to use human ACE2 (13, 15). In contrast, some studies reveal that the S protein of the *R. sinicus* SARSr-CoV with deletions (Rp3) failed to use human, civet, and bat ACE2 for cell entry (26), so we inferred that the bat SARSr-CoVs from Guangdong with RBD deletions might not be able to use human ACE2 or directly infect humans. However, whether these viruses could infect humans by ACE2 needs further investigations. Although the bat SARSr-CoV strains from Guangdong had a low risk of infecting humans because of their inability to use hACE2 at present, we should not underestimate their cross-species abilities through recombination to obtain new S genes.

In the detection of coronavirus carried by bat tissues, including heart, liver, spleen, lung, kidney, stomach, brain, anal swab, and intestine, we found that coronavirus presented only in anal swabs and intestines, indicating that this virus is transmitted through the digestive tract, in line with previous findings for SARS-CoV (27). The expression level of ACE2 in the intestines of *R. sinicus* was higher than those in other tissues, which might explain why the virus was detected in the intestine. In summary, the intestine of the bat is an important transmission route for the viruses carried by bats, which is also consistent with the research results of another study (28).

The global pandemic of SARS-CoV-2 once again reminds us to always pay attention to mutations of viruses carried by wild animals, especially bats and other vector animals, so it is necessary to monitor the coronaviruses for the long term. In our study, we found two interesting roost caves of bats in Guangzhou with high rates of coronavirus positivity and recombination. Unfortunately, we did not collect samples before 2019, so more samples are needed for in-depth study in the future. It is well known that the full length of the viral sequence can better reflect its structure and variation information, but the full length of the virus is not easy to obtain due to the low virus content in the sample and the difficulty in culturing. We will continue to pay attention to bat caves with coronaviruses, collect as many samples as possible, and try to obtain more full-length sequences of coronaviruses to comprehensively analyze the variation pattern of coronaviruses.

In conclusion, our study comprehensively analyzes the situation of coronaviruses carried by bats in Guangdong and elucidates the pattern of its transmission among bats. Meanwhile, we found that bat SARSr-CoV strains from Guangdong had a low risk of infecting humans because of their feature of being unable to use hACE2 until now, but we should not underestimate their cross-species abilities through recombination to obtain new S genes. Therefore, long-term monitoring is necessary to prevent public health incidents, and species protection provides an important reference basis.

## MATERIALS AND METHODS

### Bat sample collection and identification.

Samplings of bats were conducted from July 2008 to March 2021 in their natural roost caves in Guangdong (including Guangzhou, Huizhou, and Shenzhen) and Hainan, China (see Table S1 in the supplemental material). Bats were trapped, and anal swab samples were collected. Each sample was collected in 1 mL of viral transport medium (VTM), composed of Hanks’ balanced salt solution at pH 7.4 containing bovine serum albumin (BSA) (1%), amphotericin (15 μg/mL), and penicillin G (100 U/mL), and stored in a liquid nitrogen tank. Samples of 3 Rhinolophus sinicus and 3 *R. affinis* bats, including the heart, liver, spleen, lung, kidney, stomach, brain, and intestine, were used for quantitative real-time PCR (qRT-PCR) to determine the expression level of mRNA encoding ACE2. All samples were immediately placed into 2-mL polyethylene tubes (RNase free), stored in a liquid nitrogen tank, taken back to the laboratory, and stored at −80°C until nucleic acid extraction. Bats trapped for this study were released back into their habitat. All laboratory personnel were professionally trained and wore protective clothing to protect against biological agents before sample collection.

Bat identification was initially determined in the field by morphology (Fig. S6A) and later confirmed in the laboratory by sequencing of the mitochondrial cytochrome *b* gene (*cytb*) from samples of anal swabs. PCR amplification of the *cytb* gene was performed using DNA extracted from the swabs of bats randomly. The primers for the *cytb* gene were *cytb* F (5′-ACAGGCTCAAACAACCCAAC-3′) and *cytb* R (5′-TGGCCTCCAATTCAGGTTAG-3′). A 25-μL reaction mixture was set up, containing 12.5 μL of PCR mix (Gentech, China), 10.5 μL of double-distilled water (ddH_2_O), 1 μL of the template, 0.5 μL of the forward primer (10 μM), and 0.5 μL of the reverse primer (10 μM). Thermal cycling was performed at 94°C for 3 min, followed by 35 cycles consisting of 94°C for 30 s, 55°C for 30 s, and 72°C for 1 min and a final extension step at 72°C for 10 min. PCR products were detected on a 1.5% agarose gel and sequenced.

### Viral RNA extraction, PCR screening, and sequencing.

The anal swab samples were vortexed for 1 min, and 140 mL of the supernatant was collected from each sample after centrifugation at 3,000 rpm at 4°C for 1 min. Viral nucleic acid was extracted with a QIAamp viral RNA minikit (Qiagen, Germany) according to the manufacturer’s instructions. The tissue samples (50 mg of each sample) were ground into a powder in liquid nitrogen and transferred to a diethyl pyrocarbonate (DEPC)-treated eppendorf tubes before being volatilized by liquid nitrogen. One microgram of cDNA was synthesized using the SuperScript III first-strand synthesis system (Invitrogen, USA). RT-PCR was employed to detect the presence of coronavirus sequences using a 360-nt fragment from the S gene of bat CoV. The specific primers for the S gene were ZH2F and ZH2R (Table S2A). A 25-μL reaction mixture was set up, containing 12.5 μL of PCR mix (Gentech, China), 10.5 μL of ddH_2_O, 1 μL of the template, 0.5 μL of the forward primer (10 μM), and 0.5 μL of the reverse primer (10 μM). Thermal cycling was performed at 94°C for 3 min, followed by 35 cycles consisting of 94°C for 30 s, 55°C for 30 s, and 72°C for 1 min and a final extension step at 72°C for 10 min. PCR products were detected on a 1.5% agarose gel and sequenced.

### Metatranscriptome detection.

A total of 50 to 1,000 ng of RNA of the positive samples (Rs56) was mixed with rRNA deletion probes for rRNA depletion. Fragmentation, synthesis of 1st and 2nd strands, end repair, and adaptor ligation were conducted for library construction as described previously (29). High-throughput sequencing was conducted by the Magigene Company (Guangzhou, China). Clean reads were *de novo* assembled using MEGAHIT version 1.0 (17). BWA version 0.7.17 (30) was used to align clean reads to assembled contigs. Contigs were then classified by BLASTx against the NT database using an alignment similarity of ≥80%, a length of matched areas of ≥500 nt, and an E value of ≤10^−5^. Contigs with significant BLASTx hits were confirmed as virus sequences.

### Sequencing of full-length genomes and S genes.

The full genomic sequences of the SARSr-CoVs from positive samples (Rs56 and RaCH025) were determined by RT-PCR amplification with specific primers designed by multiple alignments of available SARS-CoV and bat SARSr-CoV sequences deposited in GenBank and additional primers from the ARTIC Network for amplifying the SARS-CoV-2 genome (https://github.com/artic-network/artic-ncov2019/blob/master/primer_schemes/nCoV-2019/V3/nCoV-2019.tsv) (Table S2A and B). The complete S genes of bat SARSr-CoVs from positive samples, with adequate amounts of RNA available, were sequenced by using primers targeted to the S genes (Table S2C). PCR products of the expected size were gel purified and subjected directly to sequencing. The sequence identity between the whole genome and different genes or regions was calculated by utilizing *p*-distance in MEGA X (31).

### Evolution analysis.

We downloaded 97 full-length genome sequences of betacoronaviruses isolated from bats from the NCBI database (https://www.ncbi.nlm.nih.gov/) (Table S3). Phylogenetic analyses were performed based on their whole-genome sequences and their ORF1ab gene, S gene, and ORF8 gene sequences. We constructed multiple-sequence alignments of their complete genomes and individual genes using MAFFT v7.407 (32). Phylogenetic analyses were estimated using MrBayes (33) with 500,000 generations and 25% of the generations as the burn-in. The best models were determined by jModelTest v2.1.7 (34). Next, the trees were visualized and exported as vector diagrams with FigTree v1.4.4 (http://tree.bio.ed.ac.uk/software/Figtree/). The aligned sequences were scanned for recombination events by the Recombination Detection Program (RDP) (35). The potential recombination events suggested by strong *P* values (<10^−20^) were further confirmed using similarity plot and bootscan analyses implemented in Simplot v3.5.1.

### Quantitative real-time PCR.

qRT-PCR was used to determine the mRNA expression levels of ACE2 in different tissues of Malayan pangolins. The total volume of the qRT-PCR mixture was 20 μL; this included 10 μL of a 2× qPCR mixture (TaKaRa, Japan), 0.5 μL of forward and reverse primers, 1 μL of the template, and remaining volume as nuclease-free water. The following procedure was used for amplification: 95°C for 30 s and 40 cycles of 95°C for 5 s, 60°C for 34 s, and 95°C for 15 s. β-Actin was used as the internal control. The primers for ACE2 of *R. sinicus* were RsACE2-F (5′-TTGCGTATGCCATGAGAGAG-3′), RsACE2-R (5′-ACAGATTTCCGGGTGAAGTG-3′), Rsβ-actin-F (5′-CTGGACTTTGAGCAGGAGATG-3′), and Rsβ-actin-R (5′-ATGGAGTTGAATGTGGTCTCG-3′). The primers for ACE2 of *R. affinis* were *Ra*ACE2-F (5′-TTCCCAAAGAGGAGTGGATG-3′), RaACE2-R (5′-ACAGGGCTTCATGAAACTGG-3′), Raβ-actin-F (5′-CTGGACTTTGAGCAGGAGATG-3′), and Raβ-actin-R (5′-GTTGAATGTGGTCTCGTGGAT-3′). The expression level of mRNA encoding ACE2 was separately determined using the comparative threshold cycle (*C_T_*) (2^−ΔΔ^*^CT^*) method. Means and standard deviations (SD) were calculated from biological replicates. Statistical significance was measured using the independent-sample *t* test by SPSS 17.0.

### Ethics statement.

The animal study was reviewed and approved by the Committee on the Ethics of Animal Experiments of the Institute of Zoology of the Guangdong Academy of Sciences.

### Data availability.

The data supporting this study are openly available at the NCBI Sequence Read Archive (SRA) and GenBank as follows: 28 sequences obtained from positive samples by RT-PCR have been deposited in GenBank under accession numbers OM263332-OM263359; S gene sequences obtained from bats (Rs56, Rs67, Rs68, Rs87, RaCH025, RaCH027, RaCH039 and Rs200609) have been deposited in GenBank under accession numbers OM222057-OM222064; metagenomics data obtained from anal swab from bat Rs56 have been deposited in SRR13787015 under BioProject accession number PRJNA705004; whole-genome sequences obtained from Rs56 and RaCH025 have been deposited in GenBank under accession numbers MW681002 and OM240725, respectively.
