# Supplementary material for: Epidemiology and Genomic Characterization of Two Novel SARS-Related Coronaviruses in Horseshoe Bats from Guangdong, China
Source: mBio. 2022 Apr 25;13(3):e00463-22. doi: 10.1128/mbio.00463-22 (PMC9239062; doi:10.1128/mbio.00463-22)
Supplement: FIG S1 [file mbio.00463-22-sf001.pdf]

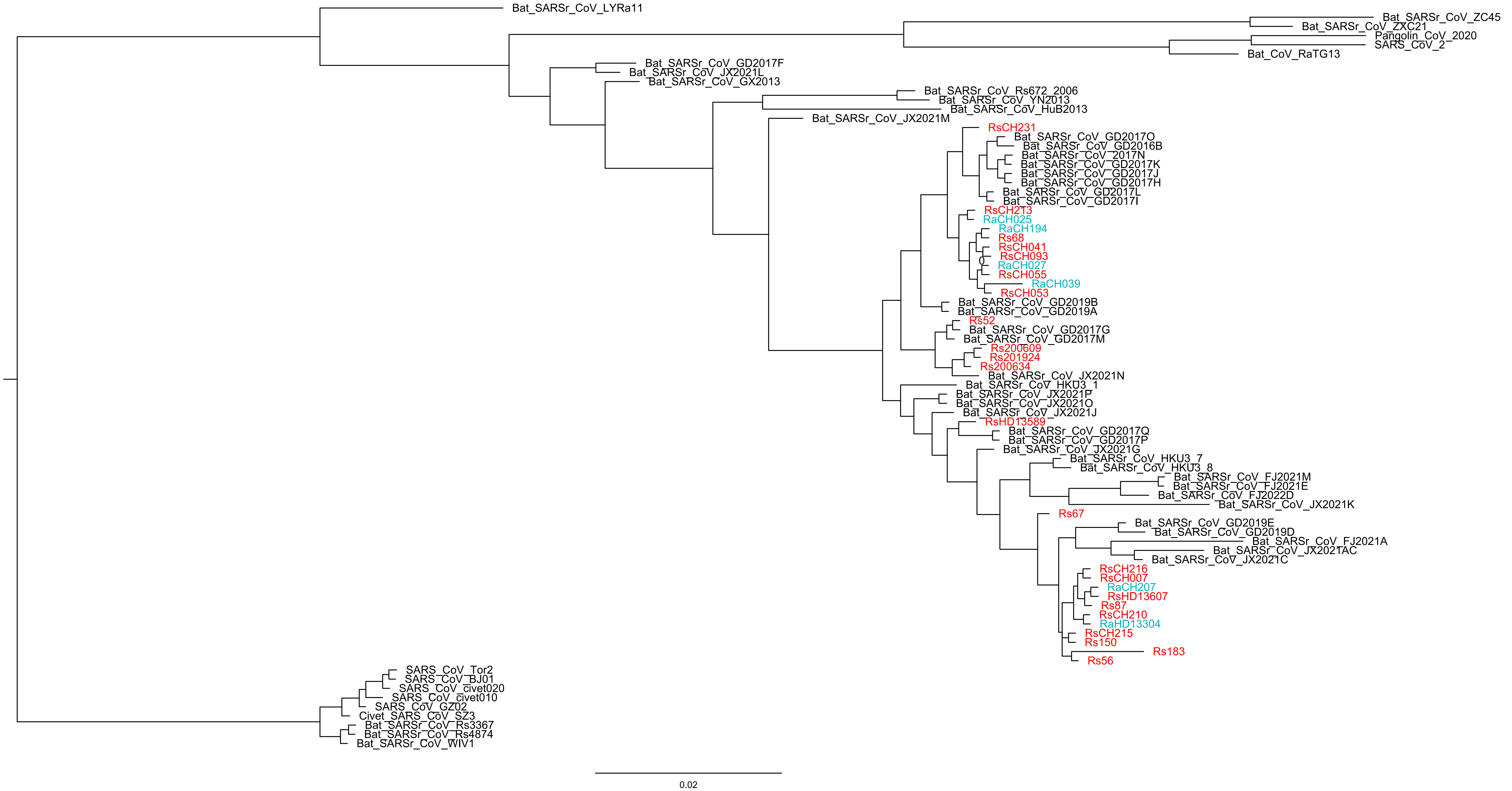

Fig. S1 Phylogenetic tree based on nucleotide sequences obtained by RT-PCR using ZH2 primers. The tree was constructed by maximum likelihood method using the MrBayes approach employing the GTR+G nucleotide substitution model. The red letters represent the SARSr-CoV strains isolated from *Rhinolophus sinicus*; The blue letters represent the SARSr-CoV strains isolated from *R. affinis*.
