# Supplementary material for: Epidemiology and Genomic Characterization of Two Novel SARS-Related Coronaviruses in Horseshoe Bats from Guangdong, China
Source: mBio. 2022 Apr 25;13(3):e00463-22. doi: 10.1128/mbio.00463-22 (PMC9239062; doi:10.1128/mbio.00463-22)
Supplement: TABLE S1 [file mbio.00463-22-st001.pdf]

Table S1 Sample information of bats

Specise	Number	Tissue	Time	Location	Result
Rhinolophus sinicus	LH12164	Anal swab	2012.10.29	Huizhou	Negative
Rhinolophus sinicus	LH12169	Anal swab	2012.10.29	Huizhou	Negative
Rhinolophus affinis	HD13296	Anal swab	2013.5.20	Huizhou	Negative
Rhinolophus sinicus	HD13297	Anal swab	2013.5.20	Huizhou	Negative
Rhinolophus affinis	HD13298	Anal swab	2013.5.20	Huizhou	Negative
Rhinolophus sinicus	HD13299	Anal swab	2013.5.20	Huizhou	Negative
Rhinolophus sinicus	HD13300	Anal swab	2013.5.20	Huizhou	Negative
Rhinolophus sinicus	HD13301	Anal swab	2013.5.20	Huizhou	Negative
Rhinolophus sinicus	HD13302	Anal swab	2013.5.20	Huizhou	Negative
Rhinolophus sinicus	HD13303	Anal swab	2013.5.20	Huizhou	Negative
Rhinolophus affinis	HD13304	Anal swab	2013.5.20	Huizhou	Positive
Rhinolophus sinicus	HD13564	Anal swab	2013.6.19	Huizhou	Negative
Rhinolophus sinicus	HD13565	Anal swab	2013.6.19	Huizhou	Negative
Rhinolophus sinicus	HD13569	Anal swab	2013.6.19	Huizhou	Negative
Rhinolophus sinicus	HD13570	Anal swab	2013.6.19	Huizhou	Negative
Rhinolophus sinicus	HD13571	Anal swab	2013.6.19	Huizhou	Negative
Rhinolophus sinicus	HD13572	Anal swab	2013.6.19	Huizhou	Negative
Rhinolophus sinicus	HD13573	Anal swab	2013.6.19	Huizhou	Negative
Rhinolophus sinicus	HD13574	Anal swab	2013.6.19	Huizhou	Negative
Rhinolophus affinis	HD13575	Anal swab	2013.6.19	Huizhou	Negative
Rhinolophus affinis	HD13576	Anal swab	2013.6.19	Huizhou	Negative
Rhinolophus sinicus	HD13577	Anal swab	2013.6.19	Huizhou	Negative
Rhinolophus affinis	HD13578	Anal swab	2013.6.19	Huizhou	Negative
Rhinolophus sinicus	HD13580	Anal swab	2013.6.19	Huizhou	Negative
Rhinolophus sinicus	HD13581	Anal swab	2013.6.19	Huizhou	Negative
Rhinolophus sinicus	HD13588	Anal swab	2013.6.19	Huizhou	Negative
Rhinolophus sinicus	HD13589	Anal swab	2013.6.19	Huizhou	Positive
Rhinolophus affinis	HD13590	Anal swab	2013.6.19	Huizhou	Negative
Rhinolophus affinis	HD13591	Anal swab	2013.6.19	Huizhou	Negative
Rhinolophus sinicus	HD13592	Anal swab	2013.6.19	Huizhou	Negative
Rhinolophus affinis	HD13593	Anal swab	2013.6.19	Huizhou	Negative
Rhinolophus sinicus	HD13594	Anal swab	2013.6.19	Huizhou	Negative
Rhinolophus sinicus	HD13595	Anal swab	2013.6.19	Huizhou	Negative
Rhinolophus affinis	HD13596	Anal swab	2013.6.19	Huizhou	Negative
Rhinolophus affinis	HD13597	Anal swab	2013.6.19	Huizhou	Negative
Rhinolophus affinis	HD13598	Anal swab	2013.6.19	Huizhou	Negative
Rhinolophus sinicus	HD13599	Anal swab	2013.6.19	Huizhou	Negative
Rhinolophus affinis	HD13600	Anal swab	2013.6.19	Huizhou	Negative
Rhinolophus affinis	HD13601	Anal swab	2013.6.19	Huizhou	Negative
Rhinolophus affinis	HD13602	Anal swab	2013.6.19	Huizhou	Negative
Rhinolophus sinicus	HD13603	Anal swab	2013.6.19	Huizhou	Negative

Rhinolophus sinicus	HD13605	Anal swab	2013.6.19	Huizhou	Negative
Rhinolophus affinis	HD13606	Anal swab	2013.6.19	Huizhou	Negative
Rhinolophus sinicus	HD13607	Anal swab	2013.6.19	Huizhou	Positive
Rhinolophus sinicus	HD13609	Anal swab	2013.6.19	Huizhou	Negative
Rhinolophus sinicus	HD13610	Anal swab	2013.6.19	Huizhou	Negative
Rhinolophus affinis	HD13611	Anal swab	2013.6.19	Huizhou	Negative
Rhinolophus affinis	HD13612	Anal swab	2013.6.19	Huizhou	Negative
Rhinolophus affinis	200596	Anal swab	2020.5.24	Huizhou	Negative
Rhinolophus affinis	200600	Anal swab	2020.5.24	Huizhou	Negative
Rhinolophus affinis	200602	Anal swab	2020.5.24	Huizhou	Negative
Rhinolophus affinis	200604	Anal swab	2020.5.24	Huizhou	Negative
Rhinolophus affinis	200607	Anal swab	2020.5.24	Huizhou	Negative
Rhinolophus affinis	200608	Anal swab	2020.5.24	Huizhou	Negative
Rhinolophus sinicus	200609	Anal swab	2020.5.24	Huizhou	Positive
Rhinolophus affinis	200613	Anal swab	2020.5.24	Huizhou	Negative
Rhinolophus affinis	200614	Anal swab	2020.5.24	Huizhou	Negative
Rhinolophus affinis	200616	Anal swab	2020.5.24	Huizhou	Negative
Rhinolophus affinis	200618	Anal swab	2020.5.24	Huizhou	Negative
Rhinolophus affinis	200619	Anal swab	2020.5.24	Huizhou	Negative
Rhinolophus affinis	200628	Anal swab	2020.5.24	Huizhou	Negative
Rhinolophus affinis	200631	Anal swab	2020.5.24	Huizhou	Negative
Rhinolophus sinicus	200634	Anal swab	2020.5.24	Huizhou	Positive
Rhinolophus affinis	200635	Anal swab	2020.5.24	Huizhou	Negative
Rhinolophus affinis	200636	Anal swab	2020.5.24	Huizhou	Negative
Rhinolophus affinis	200638	Anal swab	2020.5.24	Huizhou	Negative
Rhinolophus affinis	200639	Anal swab	2020.5.24	Huizhou	Negative
Rhinolophus affinis	201302	Anal swab	2020.6.14	Huizhou	Negative
Rhinolophus affinis	201303	Anal swab	2020.6.14	Huizhou	Negative
Rhinolophus affinis	201304	Anal swab	2020.6.14	Huizhou	Negative
Rhinolophus affinis	201305	Anal swab	2020.6.14	Huizhou	Negative
Rhinolophus affinis	201306	Anal swab	2020.6.14	Huizhou	Negative
Rhinolophus affinis	201307	Anal swab	2020.6.14	Huizhou	Negative
Rhinolophus affinis	201310	Anal swab	2020.6.14	Huizhou	Negative
Rhinolophus affinis	201311	Anal swab	2020.6.14	Huizhou	Negative
Rhinolophus affinis	201312	Anal swab	2020.6.14	Huizhou	Negative
Rhinolophus affinis	201313	Anal swab	2020.6.14	Huizhou	Negative
Rhinolophus affinis	201314	Anal swab	2020.6.14	Huizhou	Negative
Rhinolophus affinis	201315	Anal swab	2020.6.14	Huizhou	Negative
Rhinolophus affinis	201316	Anal swab	2020.6.14	Huizhou	Negative
Rhinolophus affinis	201317	Anal swab	2020.6.14	Huizhou	Negative
Rhinolophus affinis	201318	Anal swab	2020.6.14	Huizhou	Negative
Rhinolophus affinis	201319	Anal swab	2020.6.14	Huizhou	Negative
Rhinolophus affinis	201320	Anal swab	2020.6.14	Huizhou	Negative
Rhinolophus affinis	201321	Anal swab	2020.6.14	Huizhou	Negative

Rhinolophus sinicus	201322	Anal swab	2020.6.14	Huizhou	Negative
Rhinolophus affinis	201323	Anal swab	2020.6.14	Huizhou	Negative
Rhinolophus affinis	201324	Anal swab	2020.6.14	Huizhou	Negative
Rhinolophus affinis	201325	Anal swab	2020.6.14	Huizhou	Negative
Rhinolophus affinis	201326	Anal swab	2020.6.14	Huizhou	Negative
Rhinolophus affinis	201327	Anal swab	2020.6.14	Huizhou	Negative
Rhinolophus affinis	201328	Anal swab	2020.6.14	Huizhou	Negative
Rhinolophus affinis	201329	Anal swab	2020.6.14	Huizhou	Negative
Rhinolophus affinis	201330	Anal swab	2020.6.14	Huizhou	Negative
Rhinolophus affinis	201331	Anal swab	2020.6.14	Huizhou	Negative
Rhinolophus affinis	201332	Anal swab	2020.6.14	Huizhou	Negative
Rhinolophus affinis	201333	Anal swab	2020.6.14	Huizhou	Negative
Rhinolophus affinis	201334	Anal swab	2020.6.14	Huizhou	Negative
Rhinolophus affinis	201335	Anal swab	2020.6.14	Huizhou	Negative
Rhinolophus affinis	201336	Anal swab	2020.6.14	Huizhou	Negative
Rhinolophus affinis	201337	Anal swab	2020.6.14	Huizhou	Negative
Rhinolophus affinis	201339	Anal swab	2020.6.14	Huizhou	Negative
Rhinolophus affinis	201340	Anal swab	2020.6.14	Huizhou	Negative
Rhinolophus affinis	201341	Anal swab	2020.6.14	Huizhou	Negative
Rhinolophus affinis	201343	Anal swab	2020.6.14	Huizhou	Negative
Rhinolophus affinis	201344	Anal swab	2020.6.14	Huizhou	Negative
Rhinolophus affinis	201345	Anal swab	2020.6.14	Huizhou	Negative
Rhinolophus affinis	201347	Anal swab	2020.6.14	Huizhou	Negative
Rhinolophus affinis	201348	Anal swab	2020.6.14	Huizhou	Negative
Rhinolophus affinis	201349	Anal swab	2020.6.14	Huizhou	Negative
Rhinolophus affinis	201350	Anal swab	2020.6.14	Huizhou	Negative
Rhinolophus affinis	201351	Anal swab	2020.6.14	Huizhou	Negative
Rhinolophus sinicus	201921	Anal swab	2020.8.11	Huizhou	Negative
Rhinolophus sinicus	201922	Anal swab	2020.8.11	Huizhou	Negative
Rhinolophus sinicus	201923	Anal swab	2020.8.11	Huizhou	Negative
Rhinolophus sinicus	201924	Anal swab	2020.8.14	Huizhou	Positive
Rhinolophus sinicus	147	Anal swab	2009.11.8	Guangzhou	Negative
Rhinolophus sinicus	148	Anal swab	2009.11.8	Guangzhou	Negative
Rhinolophus sinicus	149	Anal swab	2009.11.8	Guangzhou	Negative
Rhinolophus sinicus	150	Anal swab	2009.11.8	Guangzhou	Positive
Rhinolophus sinicus	151	Anal swab	2009.11.8	Guangzhou	Negative
Rhinolophus sinicus	152	Anal swab	2009.11.8	Guangzhou	Negative
Rhinolophus sinicus	177	Anal swab	2009.11.8	Guangzhou	Negative
Rhinolophus sinicus	181	Anal swab	2009.11.8	Guangzhou	Negative
Rhinolophus sinicus	183	Anal swab	2009.11.8	Guangzhou	Positive
Rhinolophus sinicus	185	Anal swab	2009.11.8	Guangzhou	Negative
Rhinolophus sinicus	189	Anal swab	2009.11.8	Guangzhou	Negative
Rhinolophus sinicus	49	Anal swab	2020.4.29	Guangzhou	Negative
Rhinolophus sinicus	50	Anal swab	2020.4.29	Guangzhou	Negative

Rhinolophus sinicus	51	Anal swab	2020.4.29	Guangzhou	Negative
Rhinolophus sinicus	52	Anal swab	2020.4.29	Guangzhou	Positive
Rhinolophus sinicus	53	Anal swab	2020.4.29	Guangzhou	Negative
Rhinolophus sinicus	54	Anal swab	2020.4.29	Guangzhou	Negative
Rhinolophus sinicus	55	Anal swab	2020.4.29	Guangzhou	Negative
Rhinolophus sinicus	56	Anal swab	2020.4.29	Guangzhou	Positive
Rhinolophus sinicus	57	Anal swab	2020.4.29	Guangzhou	Negative
Rhinolophus sinicus	58	Anal swab	2020.4.29	Guangzhou	Negative
Rhinolophus sinicus	59	Anal swab	2020.4.29	Guangzhou	Negative
Rhinolophus sinicus	60	Anal swab	2020.4.29	Guangzhou	Negative
Rhinolophus sinicus	61	Anal swab	2020.4.29	Guangzhou	Negative
Rhinolophus sinicus	62	Anal swab	2020.4.29	Guangzhou	Negative
Rhinolophus sinicus	63	Anal swab	2020.4.29	Guangzhou	Negative
Rhinolophus sinicus	64	Anal swab	2020.4.29	Guangzhou	Negative
Rhinolophus sinicus	65	Anal swab	2020.4.29	Guangzhou	Negative
Rhinolophus sinicus	66	Anal swab	2020.4.29	Guangzhou	Negative
Rhinolophus sinicus	67	Anal swab	2020.4.29	Guangzhou	Positive
Rhinolophus sinicus	68	Anal swab	2020.4.29	Guangzhou	Positive
Rhinolophus sinicus	69	Anal swab	2020.4.29	Guangzhou	Negative
Rhinolophus sinicus	70	Anal swab	2020.4.29	Guangzhou	Negative
Rhinolophus sinicus	71	Anal swab	2020.4.29	Guangzhou	Negative
Rhinolophus sinicus	72	Anal swab	2020.4.29	Guangzhou	Negative
Rhinolophus sinicus	73	Anal swab	2020.4.29	Guangzhou	Negative
Rhinolophus sinicus	74	Anal swab	2020.4.29	Guangzhou	Negative
Rhinolophus sinicus	75	Anal swab	2020.4.29	Guangzhou	Negative
Rhinolophus sinicus	76	Anal swab	2020.4.29	Guangzhou	Negative
Rhinolophus sinicus	77	Anal swab	2020.4.29	Guangzhou	Negative
Rhinolophus sinicus	78	Anal swab	2020.4.29	Guangzhou	Negative
Rhinolophus sinicus	79	Anal swab	2020.4.29	Guangzhou	Negative
Rhinolophus sinicus	80	Anal swab	2020.4.29	Guangzhou	Negative
Rhinolophus sinicus	81	Anal swab	2020.4.29	Guangzhou	Negative
Rhinolophus sinicus	82	Anal swab	2020.4.29	Guangzhou	Negative
Rhinolophus sinicus	83	Anal swab	2020.4.29	Guangzhou	Negative
Rhinolophus sinicus	84	Anal swab	2020.4.29	Guangzhou	Negative
Rhinolophus sinicus	85	Anal swab	2020.4.29	Guangzhou	Negative
Rhinolophus sinicus	86	Anal swab	2020.4.29	Guangzhou	Negative
Rhinolophus sinicus	87	Anal swab	2020.4.29	Guangzhou	Positive
Rhinolophus sinicus	88	Anal swab	2020.4.29	Guangzhou	Negative
Rhinolophus sinicus	89	Anal swab	2020.4.29	Guangzhou	Negative
Rhinolophus affinis	90	Anal swab	2020.4.29	Guangzhou	Negative
Rhinolophus affinis	91	Anal swab	2020.4.29	Guangzhou	Negative
Rhinolophus affinis	92	Anal swab	2020.4.29	Guangzhou	Negative
Rhinolophus affinis	93	Anal swab	2020.4.29	Guangzhou	Negative
Rhinolophus affinis	94	Anal swab	2020.4.29	Guangzhou	Negative

Rhinolophus affinis	95	Anal swab	2020.4.29	Guangzhou	Negative
Rhinolophus affinis	96	Anal swab	2020.4.29	Guangzhou	Negative
Rhinolophus affinis	97	Anal swab	2020.4.29	Guangzhou	Negative
Rhinolophus affinis	98	Anal swab	2020.4.29	Guangzhou	Negative
Rhinolophus affinis	99	Anal swab	2020.4.29	Guangzhou	Negative
Rhinolophus affinis	100	Anal swab	2020.4.29	Guangzhou	Negative
Rhinolophus affinis	101	Anal swab	2020.4.29	Guangzhou	Negative
Rhinolophus affinis	102	Anal swab	2020.4.29	Guangzhou	Negative
Rhinolophus affinis	103	Anal swab	2020.4.29	Guangzhou	Negative
Rhinolophus affinis	104	Anal swab	2020.4.29	Guangzhou	Negative
Rhinolophus sinicus	CH001	Anal swab	2020.9.17	Guangzhou	Negative
Rhinolophus sinicus	CH003	Anal swab	2020.9.17	Guangzhou	Negative
Rhinolophus sinicus	CH005	Anal swab	2020.9.17	Guangzhou	Negative
Rhinolophus sinicus	CH007	Anal swab	2020.9.17	Guangzhou	Positive
Rhinolophus sinicus	CH011	Anal swab	2020.9.17	Guangzhou	Negative
Rhinolophus sinicus	CH013	Anal swab	2020.9.17	Guangzhou	Negative
Rhinolophus affinis	CH015	Anal swab	2020.9.17	Guangzhou	Negative
Rhinolophus affinis	CH017	Anal swab	2020.9.17	Guangzhou	Negative
Rhinolophus affinis	CH019	Anal swab	2020.9.17	Guangzhou	Negative
Rhinolophus sinicus	CH021	Anal swab	2020.9.17	Guangzhou	Negative
Rhinolophus affinis	CH023	Anal swab	2020.9.17	Guangzhou	Negative
Rhinolophus affinis	CH025	Anal swab	2020.9.17	Guangzhou	Positive
Rhinolophus affinis	CH027	Anal swab	2020.9.17	Guangzhou	Positive
Rhinolophus affinis	CH029	Anal swab	2020.9.17	Guangzhou	Negative
Rhinolophus affinis	CH031	Anal swab	2020.9.17	Guangzhou	Negative
Rhinolophus sinicus	CH033	Anal swab	2020.9.17	Guangzhou	Negative
Rhinolophus sinicus	CH035	Anal swab	2020.9.17	Guangzhou	Negative
Rhinolophus sinicus	CH037	Anal swab	2020.9.17	Guangzhou	Negative
Rhinolophus affinis	CH039	Anal swab	2020.9.17	Guangzhou	Positive
Rhinolophus sinicus	CH041	Anal swab	2020.9.17	Guangzhou	Positive
Rhinolophus sinicus	CH043	Anal swab	2020.9.17	Guangzhou	Negative
Rhinolophus affinis	CH045	Anal swab	2020.9.17	Guangzhou	Negative
Rhinolophus sinicus	CH047	Anal swab	2020.9.17	Guangzhou	Negative
Rhinolophus affinis	CH049	Anal swab	2020.9.17	Guangzhou	Negative
Rhinolophus sinicus	CH051	Anal swab	2020.9.17	Guangzhou	Negative
Rhinolophus sinicus	CH053	Anal swab	2020.9.17	Guangzhou	Positive
Rhinolophus sinicus	CH055	Anal swab	2020.9.17	Guangzhou	Positive
Rhinolophus sinicus	CH057	Anal swab	2020.9.17	Guangzhou	Negative
Rhinolophus sinicus	CH059	Anal swab	2020.9.17	Guangzhou	Negative
Rhinolophus sinicus	CH061	Anal swab	2020.9.17	Guangzhou	Negative
Rhinolophus sinicus	CH063	Anal swab	2020.9.17	Guangzhou	Negative
Rhinolophus sinicus	CH065	Anal swab	2020.9.17	Guangzhou	Negative
Rhinolophus affinis	CH067	Anal swab	2020.9.17	Guangzhou	Negative
Rhinolophus sinicus	CH069	Anal swab	2020.9.17	Guangzhou	Negative

Rhinolophus sinicus	CH071	Anal swab	2020.9.17	Guangzhou	Negative
Rhinolophus sinicus	CH073	Anal swab	2020.9.17	Guangzhou	Negative
Rhinolophus sinicus	CH075	Anal swab	2020.9.17	Guangzhou	Negative
Rhinolophus sinicus	CH077	Anal swab	2020.9.17	Guangzhou	Negative
Rhinolophus sinicus	CH079	Anal swab	2020.9.17	Guangzhou	Negative
Rhinolophus sinicus	CH081	Anal swab	2020.9.17	Guangzhou	Negative
Rhinolophus sinicus	CH083	Anal swab	2020.9.17	Guangzhou	Negative
Rhinolophus sinicus	CH085	Anal swab	2020.9.17	Guangzhou	Negative
Rhinolophus sinicus	CH087	Anal swab	2020.9.17	Guangzhou	Negative
Rhinolophus sinicus	CH089	Anal swab	2020.9.17	Guangzhou	Negative
Rhinolophus sinicus	CH091	Anal swab	2020.9.17	Guangzhou	Negative
Rhinolophus sinicus	CH093	Anal swab	2020.9.17	Guangzhou	Positive
Hipposideros pomona	CH161	Anal swab	2020.12.8	Guangzhou	Negative
Hipposideros pomona	CH162	Anal swab	2020.12.8	Guangzhou	Negative
Hipposideros pomona	CH163	Anal swab	2020.12.8	Guangzhou	Negative
Hipposideros pomona	CH164	Anal swab	2020.12.8	Guangzhou	Negative
Hipposideros pomona	CH165	Anal swab	2020.12.8	Guangzhou	Negative
Hipposideros pomona	CH166	Anal swab	2020.12.8	Guangzhou	Negative
Hipposideros pomona	CH167	Anal swab	2020.12.8	Guangzhou	Negative
Hipposideros pomona	CH168	Anal swab	2020.12.8	Guangzhou	Negative
Rhinolophus affinis	CH169	Anal swab	2021.3.20	Guangzhou	Negative
Rhinolophus affinis	CH170	Anal swab	2021.3.20	Guangzhou	Negative
Rhinolophus affinis	CH171	Anal swab	2021.3.20	Guangzhou	Negative
Rhinolophus affinis	CH172	Anal swab	2021.3.20	Guangzhou	Negative
Rhinolophus affinis	CH173	Anal swab	2021.3.20	Guangzhou	Negative
Rhinolophus affinis	CH174	Anal swab	2021.3.20	Guangzhou	Negative
Rhinolophus affinis	CH175	Anal swab	2021.3.20	Guangzhou	Negative
Rhinolophus affinis	CH176	Anal swab	2021.3.20	Guangzhou	Negative
Rhinolophus affinis	CH177	Anal swab	2021.3.20	Guangzhou	Negative
Rhinolophus affinis	CH178	Anal swab	2021.3.20	Guangzhou	Negative
Rhinolophus affinis	CH179	Anal swab	2021.3.20	Guangzhou	Negative
Rhinolophus affinis	CH180	Anal swab	2021.3.20	Guangzhou	Negative
Rhinolophus affinis	CH181	Anal swab	2021.3.20	Guangzhou	Negative
Rhinolophus affinis	CH182	Anal swab	2021.3.20	Guangzhou	Negative
Rhinolophus affinis	CH183	Anal swab	2021.3.20	Guangzhou	Negative
Rhinolophus affinis	CH184	Anal swab	2021.3.20	Guangzhou	Negative
Rhinolophus affinis	CH185	Anal swab	2021.3.20	Guangzhou	Negative
Rhinolophus affinis	CH186	Anal swab	2021.3.20	Guangzhou	Negative
Rhinolophus affinis	CH187	Anal swab	2021.3.20	Guangzhou	Negative
Rhinolophus affinis	CH188	Anal swab	2021.3.20	Guangzhou	Negative
Rhinolophus affinis	CH189	Anal swab	2021.3.20	Guangzhou	Negative
Rhinolophus affinis	CH190	Anal swab	2021.3.20	Guangzhou	Negative
Rhinolophus affinis	CH191	Anal swab	2021.3.20	Guangzhou	Negative
Rhinolophus affinis	CH192	Anal swab	2021.3.20	Guangzhou	Negative

Rhinolophus affinis	CH193	Anal swab	2021.3.20	Guangzhou	Negative
Rhinolophus affinis	CH194	Anal swab	2021.3.20	Guangzhou	Positive
Rhinolophus affinis	CH195	Anal swab	2021.3.20	Guangzhou	Negative
Rhinolophus affinis	CH196	Anal swab	2021.3.20	Guangzhou	Negative
Rhinolophus affinis	CH197	Anal swab	2021.3.20	Guangzhou	Negative
Rhinolophus sinicus	CH198	Anal swab	2021.3.20	Guangzhou	Negative
Rhinolophus affinis	CH199	Anal swab	2021.3.20	Guangzhou	Negative
Rhinolophus affinis	CH200	Anal swab	2021.3.20	Guangzhou	Negative
Rhinolophus affinis	CH201	Anal swab	2021.3.20	Guangzhou	Negative
Rhinolophus affinis	CH202	Anal swab	2021.3.20	Guangzhou	Negative
Rhinolophus affinis	CH203	Anal swab	2021.3.20	Guangzhou	Negative
Rhinolophus affinis	CH204	Anal swab	2021.3.20	Guangzhou	Negative
Rhinolophus affinis	CH205	Anal swab	2021.3.20	Guangzhou	Negative
Rhinolophus affinis	CH206	Anal swab	2021.3.20	Guangzhou	Negative
Rhinolophus affinis	CH207	Anal swab	2021.3.20	Guangzhou	Positive
Rhinolophus sinicus	CH208	Anal swab	2021.3.20	Guangzhou	Negative
Rhinolophus sinicus	CH209	Anal swab	2021.3.20	Guangzhou	Negative
Rhinolophus sinicus	CH210	Anal swab	2021.3.20	Guangzhou	Positive
Rhinolophus sinicus	CH211	Anal swab	2021.3.20	Guangzhou	Negative
Rhinolophus sinicus	CH212	Anal swab	2021.3.20	Guangzhou	Negative
Rhinolophus sinicus	CH213	Anal swab	2021.3.20	Guangzhou	Positive
Rhinolophus sinicus	CH214	Anal swab	2021.3.20	Guangzhou	Negative
Rhinolophus sinicus	CH215	Anal swab	2021.3.20	Guangzhou	Positive
Rhinolophus sinicus	CH216	Anal swab	2021.3.20	Guangzhou	Positive
Rhinolophus sinicus	CH217	Anal swab	2021.3.20	Guangzhou	Negative
Rhinolophus sinicus	CH218	Anal swab	2021.3.20	Guangzhou	Negative
Hipposideros pomona	CH219	Anal swab	2021.3.20	Guangzhou	Negative
Rhinolophus sinicus	CH220	Anal swab	2021.3.20	Guangzhou	Negative
Rhinolophus affinis	CH221	Anal swab	2021.3.20	Guangzhou	Negative
Rhinolophus sinicus	CH222	Anal swab	2021.3.20	Guangzhou	Negative
Rhinolophus sinicus	CH223	Anal swab	2021.3.20	Guangzhou	Negative
Rhinolophus affinis	CH224	Anal swab	2021.3.20	Guangzhou	Negative
Rhinolophus sinicus	CH225	Anal swab	2021.3.20	Guangzhou	Negative
Rhinolophus sinicus	CH226	Anal swab	2021.3.20	Guangzhou	Negative
Rhinolophus sinicus	CH227	Anal swab	2021.3.20	Guangzhou	Negative
Rhinolophus sinicus	CH228	Anal swab	2021.3.20	Guangzhou	Negative
Rhinolophus sinicus	CH229	Anal swab	2021.3.20	Guangzhou	Negative
Rhinolophus sinicus	CH230	Anal swab	2021.3.20	Guangzhou	Negative
Rhinolophus sinicus	CH231	Anal swab	2021.3.20	Guangzhou	Positive
Rhinolophus sinicus	CH232	Anal swab	2021.3.20	Guangzhou	Negative
Rhinolophus sinicus	CH233	Anal swab	2021.3.20	Guangzhou	Negative
Rhinolophus affinis	CH234	Anal swab	2021.3.20	Guangzhou	Negative
Rhinolophus sinicus	CH235	Anal swab	2021.3.20	Guangzhou	Negative
Hipposideros pomona	CH236	Anal swab	2021.3.20	Guangzhou	Negative

Rhinolophus sinicus	CH237	Anal swab	2021.3.20	Guangzhou	Negative
Rhinolophus sinicus	CH238	Anal swab	2021.3.20	Guangzhou	Negative
Rhinolophus sinicus	CH239	Anal swab	2021.3.20	Guangzhou	Negative
Rhinolophus sinicus	CH240	Anal swab	2021.3.20	Guangzhou	Negative
Hipposideros pomona	CH241	Anal swab	2021.3.20	Guangzhou	Negative
Rhinolophus sinicus	CH242	Anal swab	2021.3.20	Guangzhou	Negative
Rhinolophus sinicus	CH243	Anal swab	2021.3.20	Guangzhou	Negative
Rhinolophus sinicus	210520	Anal swab	2021.5.22	Guangzhou	Negative
Rhinolophus sinicus	210521	Anal swab	2021.5.22	Guangzhou	Negative
Rhinolophus affinis	210522	Anal swab	2021.5.22	Guangzhou	Negative
Rhinolophus affinis	210523	Anal swab	2021.5.22	Guangzhou	Negative
Rhinolophus affinis	210524	Anal swab	2021.5.22	Guangzhou	Negative
Rhinolophus affinis	210525	Anal swab	2021.5.22	Guangzhou	Negative
Rhinolophus affinis	210526	Anal swab	2021.5.22	Guangzhou	Negative
Rhinolophus affinis	210527	Anal swab	2021.5.22	Guangzhou	Negative
Rhinolophus affinis	210528	Anal swab	2021.5.22	Guangzhou	Negative
Rhinolophus affinis	210529	Anal swab	2021.5.22	Guangzhou	Negative
Rhinolophus affinis	210530	Anal swab	2021.5.22	Guangzhou	Negative
Rhinolophus affinis	210531	Anal swab	2021.5.22	Guangzhou	Negative
Rhinolophus affinis	210532	Anal swab	2021.5.22	Guangzhou	Negative
Rhinolophus affinis	210533	Anal swab	2021.5.22	Guangzhou	Negative
Rhinolophus affinis	210534	Anal swab	2021.5.22	Guangzhou	Negative
Rhinolophus sinicus	210535	Anal swab	2021.5.22	Guangzhou	Negative
Rhinolophus affinis	210536	Anal swab	2021.5.22	Guangzhou	Negative
Rhinolophus affinis	210537	Anal swab	2021.5.22	Guangzhou	Negative
Rhinolophus affinis	210538	Anal swab	2021.5.22	Guangzhou	Negative
Rhinolophus affinis	210539	Anal swab	2021.5.22	Guangzhou	Negative
Rhinolophus sinicus	SZ095	Anal swab	2020.9.20	Shenzhen	Negative
Rhinolophus sinicus	SZ097	Anal swab	2020.9.20	Shenzhen	Negative
Rhinolophus sinicus	SZ099	Anal swab	2020.9.20	Shenzhen	Negative
Rhinolophus sinicus	SZ101	Anal swab	2020.9.20	Shenzhen	Negative
Rhinolophus sinicus	SZ103	Anal swab	2020.9.20	Shenzhen	Negative
Rhinolophus sinicus	SZ105	Anal swab	2020.9.20	Shenzhen	Negative
Rhinolophus sinicus	SZ107	Anal swab	2020.9.20	Shenzhen	Negative
Rhinolophus sinicus	SZ109	Anal swab	2020.9.20	Shenzhen	Negative
Rhinolophus sinicus	SZ111	Anal swab	2020.9.20	Shenzhen	Negative
Rhinolophus sinicus	SZ113	Anal swab	2020.9.20	Shenzhen	Negative
Rhinolophus sinicus	SZ115	Anal swab	2020.9.20	Shenzhen	Negative
Rhinolophus affinis	SZ117	Anal swab	2020.9.21	Shenzhen	Negative
Rhinolophus affinis	SZ119	Anal swab	2020.9.21	Shenzhen	Negative
Rhinolophus affinis	SZ121	Anal swab	2020.9.21	Shenzhen	Negative
Rhinolophus affinis	SZ123	Anal swab	2020.9.21	Shenzhen	Negative
Rhinolophus affinis	SZ129	Anal swab	2020.9.21	Shenzhen	Negative
Rhinolophus sinicus	SZ131	Anal swab	2020.9.21	Shenzhen	Negative

Rhinolophus sinicus	SZ133	Anal swab	2020.9.21	Shenzhen	Negative
Rhinolophus sinicus	SZ139	Anal swab	2020.9.21	Shenzhen	Negative
Rhinolophus affinis	SZ141	Anal swab	2020.9.21	Shenzhen	Negative
Rhinolophus affinis	SZ143	Anal swab	2020.9.21	Shenzhen	Negative
Rhinolophus affinis	SZ145	Anal swab	2020.9.21	Shenzhen	Negative
Rhinolophus affinis	SZ147	Anal swab	2020.9.21	Shenzhen	Negative
Rhinolophus affinis	SZ149	Anal swab	2020.9.21	Shenzhen	Negative
Rhinolophus affinis	SZ153	Anal swab	2020.9.21	Shenzhen	Negative
Rhinolophus affinis	SZ155	Anal swab	2020.9.21	Shenzhen	Negative
Rhinolophus sinicus	SZ157	Anal swab	2020.9.21	Shenzhen	Negative
Rhinolophus affinis	SZ159	Anal swab	2020.9.21	Shenzhen	Negative
Rhinolophus sinicus	YG07	Anal swab	2008.7.2	Hainan	Negative
Rhinolophus sinicus	YG11	Anal swab	2008.7.2	Hainan	Negative
Rhinolophus sinicus	YG14	Anal swab	2008.7.2	Hainan	Negative
Rhinolophus sinicus	YG16	Anal swab	2008.7.2	Hainan	Negative
Rhinolophus sinicus	YG18	Anal swab	2008.7.2	Hainan	Negative
Rhinolophus sinicus	YG19	Anal swab	2008.7.2	Hainan	Negative
Rhinolophus sinicus	YG20	Anal swab	2008.7.2	Hainan	Negative
Rhinolophus sinicus	YG21	Anal swab	2008.7.2	Hainan	Negative
Rhinolophus sinicus	YG22	Anal swab	2008.7.2	Hainan	Negative
Rhinolophus sinicus	YG23	Anal swab	2008.7.2	Hainan	Negative
Rhinolophus sinicus	CX01	Anal swab	2008.7.5	Hainan	Negative
Rhinolophus sinicus	CX03	Anal swab	2008.7.5	Hainan	Negative
Rhinolophus sinicus	CX04	Anal swab	2008.7.5	Hainan	Negative
Rhinolophus sinicus	CX05	Anal swab	2008.7.5	Hainan	Negative
Rhinolophus sinicus	CX06	Anal swab	2008.7.5	Hainan	Negative
Rhinolophus sinicus	CX07	Anal swab	2008.7.5	Hainan	Negative
Rhinolophus sinicus	CX08	Anal swab	2008.7.5	Hainan	Negative
Rhinolophus sinicus	CX09	Anal swab	2008.7.5	Hainan	Negative
Rhinolophus sinicus	CX10	Anal swab	2008.7.5	Hainan	Negative
Rhinolophus sinicus	CX11	Anal swab	2008.7.5	Hainan	Negative
Rhinolophus sinicus	CX12	Anal swab	2008.7.5	Hainan	Negative
Rhinolophus sinicus	XL26	Anal swab	2008.7.6	Hainan	Negative
Rhinolophus sinicus	XL31	Anal swab	2008.7.6	Hainan	Negative
Rhinolophus sinicus	XL32	Anal swab	2008.7.6	Hainan	Negative
Rhinolophus sinicus	MG04	Anal swab	2008.7.21	Hainan	Negative
Rhinolophus sinicus	MG07	Anal swab	2008.7.21	Hainan	Negative
Rhinolophus sinicus	xx01	Anal swab	2008.7.23	Hainan	Negative
Rhinolophus sinicus	xx02	Anal swab	2008.7.23	Hainan	Negative
Rhinolophus sinicus	xx03	Anal swab	2008.7.23	Hainan	Negative
