# Supplementary material for: Epidemiology and Genomic Characterization of Two Novel SARS-Related Coronaviruses in Horseshoe Bats from Guangdong, China
Source: mBio. 2022 Apr 25;13(3):e00463-22. doi: 10.1128/mbio.00463-22 (PMC9239062; doi:10.1128/mbio.00463-22)
Supplement: FIG S2 [file mbio.00463-22-sf002.pdf]

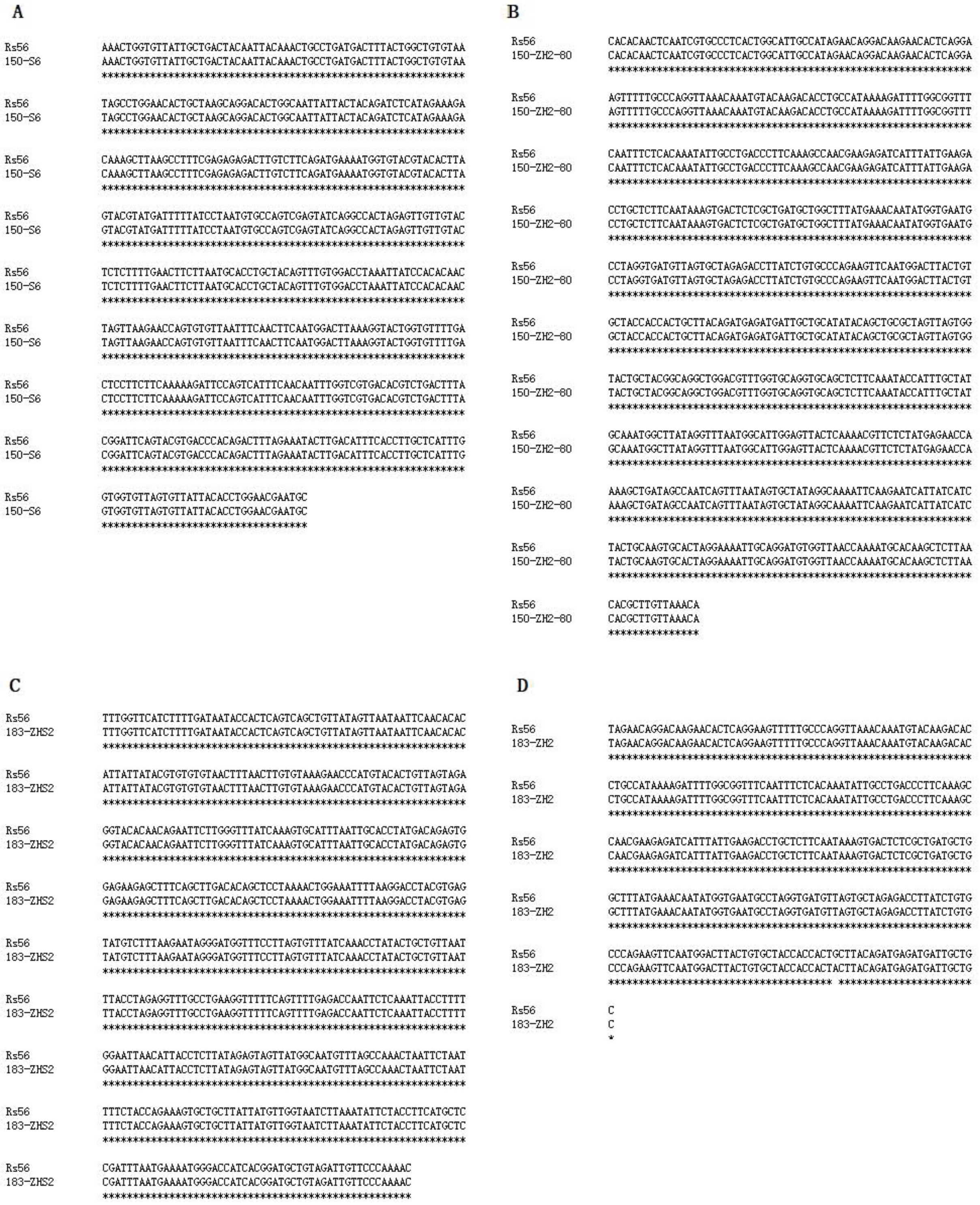

Fig. S2 Sequence alignment of SARSr-CoV from Guangdong in 2009 and 2020. (A) Sequence alignment between Rs150 and Rs56 using the prime S6. (B) Sequence alignment between Rs150 and Rs56 using the prime ZH2 and 80. (C) Sequence alignment between Rs183 and Rs56 using the prime ZHS2. (D) Sequence alignment between Rs183 and Rs56 using the prime ZH2.
