# Supplementary material for: Epidemiology and Genomic Characterization of Two Novel SARS-Related Coronaviruses in Horseshoe Bats from Guangdong, China
Source: mBio. 2022 Apr 25;13(3):e00463-22. doi: 10.1128/mbio.00463-22 (PMC9239062; doi:10.1128/mbio.00463-22)
Supplement: FIG S3 [file mbio.00463-22-sf003.pdf]

```
Rs56       MKILILAFLASLAKAQEGCGIISRKPQPKMAQVSSSRRGVYYNDDIFRSDVLHLTQDYFL
Rs87       MKILILAFLASLAKAQEGCGIISRKPQPKMAQVSSSRRGVYYNDDIFRSDVLHLTQDYFL
Rs68       MKILILAFLASLAKAQEGCGIISRKPQPKMAQVSSSRRGVYYNDDIFRSDVLHLTQDYFL
RaCH025    MKILILAFLASLAKAQEGCGIISRKPQPKMAQVSSSRRGVYYNDDIFRSDVLHLTQDYFL
RaCH039    MKILILAFLASLAKAQEGCGIISRKPQPKMAQVSSSRRGVYYNDDIFRSDVLHLTQDYFL
RaCH027    MKILILAFLASLAKAQEGCGIISRKPQPKMAQVSSSRRGVYYNDDIFRSDVLHLTQDYFL
Rs67       MKILILAFLASLAKAQEGCGIISRKPQPKMAQVSSSRRGVYYNDDIFRSDVLHLTQDYFL
Rs200609   MKILILAFLASLAKAQEGCGIISRKQQPKMAQVSSSRRGVYYNDDIFRSDVLHLTQDYFL
           ************************ ***********************************
```

```
Rs56       PFDSNLTQYFSLNVDSDRFTYFDNPILNFGDGVYFAATEKSNVIRGWIFGSSFDNTTQSA
Rs87       PFDSNLTQYFSLNVDSDRFTYFDNPILNFGDGVYFAATEKSNVIRGWIFGSSFDNTTQSA
Rs68       PFDSNLTQYFSLNVDSDRFTYFDNPILNFGDGVYFAATEKSNVIRGWIFGSSFDNTTQSA
RaCH025    PFDSNLTQYFSLNVDSDRFTYFDNPILNFGDGVYFAATEKSNVIRGWIFGSSFDNTTQSA
RaCH039    PFDSNLTQYFSLNVDSDRFTYFDNPILNFGDGVYFAATEKSNVIRGWIFGSSFDNTTQSA
RaCH027    PFDSNLTQYFSLNVDSDRFTYFDNPILNFGDGVYFAATEKSNVIRGWIFGSSFDNTTQSA
Rs67       PFDSNLTQYFSLNVDSDRFTYFDNPILNFGDGVYFAATEKSNVIRGWIFGSSFDNTTQSA
Rs200609   PXXXXXXXXXXXXXXXXXXXXXXXXXXXXXXXXXXXXXXXXXXXXXXXXXXXXXXXXXSA
           *                                                         **
```

```
Rs56       VIVNNSTHIIIRVCNFNLCKEPMYTVSRGTQQNSWVYQSAFNCTYDRVEKSFQLDTAPKT
Rs87       VIVNNSTHIIIRVCNFNLCKEPMYTVSRGTQQNSWVYQSAFNCTYDRVEKSFQLDTAPKT
Rs68       VIVNNSTHIIIRVCNFNLCKEPMYTVSRGTQQNSWVYQSAFNCTYDRVEKSFQLDTAPKT
RaCH025    VIVNNSTHIIIRVCNFNLCKEPMYTVSRGTQQNSWVYQSAFNCTYDRVEKSFQLDTAPKT
RaCH039    VIVNNSTHIIIRVCNFNLCKEPMYTVSRGTQQNSWVYQSAFNCTYDRVEKSFQLDTAPKT
RaCH027    VIVNNSTHIIIRVCNFNLCKEPMYTVSRGTQQNSWVYQSAFNCTYDRVEKSFQLDTAPKT
Rs67       VIVNNSTHIIIRVCNFNLCKEPMYTVSRGTQQNSWVYQSAFNCTYDRVEKSFQLDTAPKT
Rs200609   VIVNNSTHIIIRVCNFNLCKEPMYTVSRGTQQNSWVYQSAFNCTYDRVEKSFQLDNSPKT
           ******************************************************.:****
```

```
Rs56       GNFKDLREYVFKNRDGFLSVYQTYTAVNLPRGLPEGFSVLRPILKLPFGINITSYRVVMA
Rs87       GNFKDLREYVFKNRDGFLSVYQTYTAVNLPRGLPEGFSVLRPILKLPFGINITSYRVVMA
Rs68       GNFKDLREYVFKNRDGFLSVYQTYTAVNLPRGLPEGFSVLRPILKLPFGINITSYRVVMA
RaCH025    GNFKDLREYVFKNRDGFLSVYQTYTAVNLPRGLPEGFSVLRPILKLPFGINITSYRVVMA
RaCH039    GNFKDLREYVFKNRDGFLSVYQTYTAVNLPRGLPEGFSVLRPILKLPFGINITSYRVVMA
RaCH027    GNFKDLREYVFKNRDGFLSVYQTYTAVNLPRGLPEGFSVLRPILKLPFGINITSYRVVMA
Rs67       GNFKDLREYVFKNRDGFLSVYQTYTAVNLPRGLPEGFSVLRPILKLPFGINITSYRVVMA
Rs200609   GNFKDLREYVFKNKDGFLSVYQTYTAIDIIRGLPEGFSVLRPILKLPFGINITSYRVVMA
           *************:************.:: ******************************
```

```
Rs56       MFSQTNSNFLPESAAYYVGNLKYSTFMLRFNENGTITDAVDCSQNPLAELKCTIKNFKVD
Rs87       MFSQTNSNFLPESAAYYVGNLKYSTFMLRFNENGTITDAVDCSQNPLAELKCTIKNFKVD
Rs68       MFSQTNSNFLPESAAYYVGNLKYSTFMLRFNENGTITDAVDCSQNPLAELKCTIKNFKVD
RaCH025    MFSQTNSNFLPESAAYYVGNLKYSTFMLRFNENGTITDAVDCSQNPLAELKCTIKNFKVD
RaCH039    MFSQTNSNFLPESAAYYVGNLKYSTFMLRFNENGTITDAVDCSQNPLAELKCTIKNFKVD
RaCH027    MFSQTNSNFLPESAAYYVGNLKYSTFMLRFNENGTITDAVDCSQNPLAELKCTIKNFKVD
Rs67       MFSQINSNFLPESAAYYVGNLKYSTFMLRFNENGTITDAVDCSQNPLAELKCTIKNFKVD
Rs200609   MFSQTTSNFLPESAAYYVGNLKYSTFMLRFNENGTITDAVDCSQNPLAEXXXXXXXXXXX
           **** .*******************************************
```

```
Rs56       KGIYQTSNFRVSPTQEVIRFPNITNLCPFDKVFNATRFPNVYAWERTKISDCVADYTVLY
Rs87       KGIYQTSNFRVSPTQEVIRFPNITNLCPFDKVFNATRFPNVYAWERTKISDCVADYTVLY
Rs68       KGIYQTSNFRVSPTQEVIRFPNITNLCPFDKVFNATRFPNVYAWERTKISDCVADYTVLY
RaCH025    KGIYQTSNFRVSPTQEVIRFPNITNLCPFDKVFNATRFPNVYAWERTKISDCVADYTVLY
RaCH039    KGIYQTSNFRVSPTQEVIRFPNITNLCPFDKVFNATRFPNVYAWERTKISDCVADYTVLY
RaCH027    KGIYQTSNFRVSPTQEVIRFPNITNLCPFDKVFNATRFPNVYAWERTKISDCVADYTVLY
Rs67       KGIYQTSNFRVSPTQEVIRFPNITNLCPFDKVFNATRFPNVYAWERTKISDCVADYTVLY
Rs200609   XXXXXXXXXXXXXXXXXXXXXXXXXXXXXXXXXXXXXXXXXXXXXXXXXXXXXXXXXXXX
```

```
Rs56       NSTSFSTFKCYGVSPSKLIDLCFTSVYADTFLIRSSEVRQIAPGETGVIADYNYKLPDDF
Rs87       NSTSFSTFKCYGVSPSKLIDLCFTSVYADTFLIRSSEVRQIAPGETGVIADYNYKLPDDF
Rs68       NSTSFSTFKCYGVSPSKLIDLCFTSVYADTFLIRSSEVRQIAPGETGVIADYNYKLPDDF
RaCH025    NSTSFSTFKCYGVSPSKLIDLCFTSVYADTFLIRSSEVRQIAPGETGVIADYNYKLPDDF
RaCH039    NSTSFSTFKCYGVSPSKLIDLCFTSVYADTFLIRSSEVRQIAPGETGVIADYNYKLPDDF
RaCH027    NSTSFSTFKCYGVSPSKLIDLCFTSVYADTFLIRSSEVRQIAPGETGVIADYNYKLPDDF
Rs67       NSTSFSTFKCYGVSPSKLIDLCFTSVYADTFLIRSSEVRQIAPGETGVIADYNYKLPDDF
Rs200609   XXXXXXXXXXXXXXXXXXXXXXXXXXXXXXXXXXXXXXXXXXPGETGVIADYNYKLPDDF
                                                     ******************
```

```
Rs56       TGCVIAWNTAKQDTGNYYYRSHRKTKLKPFERDLSSDE-NGVRTLSTYDFYPNVPVEYQA
Rs87       TGCVIAWNTAKQDTGNYYYRSHRKTKLKPFERDLSSDE-NGVRTLSTYDFYPNVPVEYQA
Rs68       TGCVIAWNTAKQDTGNYYYRSHRKTKLKPFERDLSSDE-NGVRTLSTYDFYPNVPVEYQA
RaCH025    TGCVIAWNTAKQDTGNYYYRSHRKTKLKPFERDLSSDE-NGVRTLSTYDFYPNVPVEYQA
RaCH039    TGCVIAWNTAKQDTGNYYYRSHRKTKLKPFERDLSSDE-NGVRTLSTYDFYPNVPVEYQA
RaCH027    TGCVIAWNTAKQDTGNYYYRSHRKTKLKPFERDLSSDE-NGVRTLSTYDFYSNVPVEYQA
Rs67       TGCVIAWNTAKQDTGNYYYRSHRKTKLKPFERDLSSDE-NGVRTLSTYDFYPNVPVEYQA
Rs200609   TGCVIAWNTAKQDTGNYYYRSHRKTKLKPFERDLSSDDGNGVYTLSTYDFNPNVPVAYQA
           *************************************: *** *******.***** ***
```

```
Rs56       TRVVVLSFELLNAPATVCGPKLSTQLVKNQCVNFNFNGLKGTGVLTPSSKRFQSFQQFGR
Rs87       TRVVVLSFELLNAPATVCGPKLSTQLVKNQCVNFNFNGLKGTGVLTPSSKRFQSFQQFGR
Rs68       TRVVVLSFELLNAPATVCGPKLSTQLVKNQCVNFNFNGLKGTGVLTPSSKRFQSFQQFGR
RaCH025    TRVVVLSFELLNAPATVCGPKLSTQLVKNQCVNFNFNGLKGTGVLTPSSKRFQSFQQFGR
RaCH039    TRVVVLSFELLNAPATVCGPKLSTQLVKNQCVNFNFNGLKGTGVLTPSSKRFQSFQQFGR
RaCH027    TRVVVLSFELLNAPATVCGPKLSTQLVKNQCVNFNFNGLKGTGVLTPSSKRFQSFQQFGR
```

```
Rs67        TRVVVLSFELLNAPATVCGPKLSTQLVKNQCVNFNFNGLKGTGVLTPSSKRFQSFQQFGR
Rs200609    TRVVVLSFELLNAPATVCGPKLSTQLVKNQCVNFNFNGLKGTGVLTPSSKRFQSFQQFGR
            ************************************************************

Rs56        DTSDFTDSVRDPQTLEILDISPCSFGGVSVITPGTNASSEVAVLYQDVNCTDVPTAIRAD
Rs87        DTSDFTDSVRDPQTLEILDISPCSFGGVSVITPGTNASSEVAVLYQDVNCTDVPTAIRAD
Rs68        DTSDFTDSVRDPQTLEILDISPCSFGGVSVITPGTNASSEVAVLYQDVNCTDVPTAIRAD
RaCH025     DTSDFTDSVRDPQTLEILDISPCSFGGVSVITPGTNASSEVAVLYQDVNCTDVPTAIRAD
RaCH039     DTSDFTDSVRDPQTLEILDISPCSFGGVSVITPGTNASSEVAVLYQDVNCTDVPTAIRAD
RaCH027     DTSDFTDSVRDPQTLEILDISPCSFGGVSVITPGTNASSEVAVLYQDVNCTDVPTAIRAD
Rs67        DTSDFTDSVRDPQTLEILDISPCSFGGVSVITPGTNASSEVAVLYQDVNCTDVPTAIRAD
Rs200609    DTSDFTDSVRDPQTLEILDISPCSFGGVSVITPGTNASSEVAVLYQDVNCTDVPTAIRAD
            ************************************************************

Rs56        QLTPAWRVYSTGVNVFQTQAGCLIGAEHVNASYECDIPIGAGICASYHTASVLRSTGQKS
Rs87        QLTPAWRVYSTGVNVFQTQAGCLIGAEHVNASYECDIPIGAGICASYHTASVLRSTGQKS
Rs68        QLTPAWRVYSTGVNVFQTQAGCLIGAEHVNASYECDIPIGAGICASYHTASVLRSTGQKS
RaCH025     QLTPAWRVYSTGVNVFQTQAGCLIGAEHVNASYECDIPIGAGICASYHTASVLRSTGQKS
RaCH039     QLTPAWRVYSTGVNVFQTQAGCLIGAEHVNASYECDIPIGAGICASYHTASVLRSTGQKS
RaCH027     QLTPAWRVYSTGVNVFQTQAGCLIGAEHVNASYECDIPIGAGICASYHTASVLRSTGQKS
Rs67        QLTPAWRVYSTGVNVFQTQAGCLIGAEHVNASYECDIPIGAGICASYHTASVLRSTGQKS
Rs200609    QLTPAWRVYSTGVNVFQTQAGCLIGAEHVNASYECDIPIGAGVCASYHTASVLRSTGQKS
            *******************************************:****************

Rs56        IVAYTMSLGAENSIAYANNSIAIPTNFSISVTTEVMPVSMAKTSVDCTMYICGDSLECSN
Rs87        IVAYTMSLGAENSIAYANNSIAIPTNFSISVTTEVMPVSMAKTSVDCTMYICGDSLECSN
Rs68        IVAYTMSLGAENSIAYANNSIAIPTNFSISVTTEVMPVSMAKTSVDCTMYICGDSLECSN
RaCH025     IVAYTMSLGAENSIAYANNSIAIPTNFSISVTTEVMPVSMAKTSVDCTMYICGDSLECSN
RaCH039     IVAYTMSLGAENSIAYANNSIAIPTNFSISVTTEVMPVSMAKTSVDCTMYICGDSLECSN
RaCH027     IVAYTMSLGAENSIAYANNSIAIPTNFSISVTTEVMPVSMAKTSVDCTMYICGDSLECSN
Rs67        IVAYTMSLGAENSIAYANNSIAIPTNFSISVTTEVMPVSMAKTSVDCTMYICGDSLECSN
Rs200609    IVAYTMSLGAENSIAYANNSIAIPTNFSISVTTEVMPVSMAKTSVDCTMYICGDSLECSN
            ************************************************************

Rs56        LLLQYGSFCTQLNRALTGIAIEQDKNTQEVFAQVKQMYKTPAIKDFGGFNFSQILPDPSK
Rs87        LLLQYGSFCTQLNRALTGIAIEQDKNTQEVFAQVKQMYKTPAIKDFGGFNFSQILPDPSK
Rs68        LLLQYGSFCTQLNRALTGIAIEQDKNTQEVFAQVKQMYKTPAIKDFGGFNFSQILPDPSK
RaCH025     LLLQYGSFCTQLNRALTGIAIEQDKNTQEVFAQVKQMYKTPAIKDFGGFNFSQILPDPSK
RaCH039     LLLQYGSFCTQLNRALTGIAIEQDKNTQEVFAQVKQMYKTPAIKDFGGFNFSQILPDPSK
RaCH027     LLLQYGSFCTQLNRALTGIAIEQDKNTQEVFAQVKQMYKTPAIKDFGGFNFSQILPDPSK
Rs67        LLLQYGSFCTQLNRALTGIAIEQDKNTQEVFAQVKQMYKTPAIKDFGGFNFSQILPDPSK
Rs200609    LLLQYGSFCTQLNRALTGIAIEQDKNTQEVFAQVKQMYKTPAIKDFGGFNFSQILPDPSK
            ************************************************************

Rs56        PTKRSFIEDLLFNKVTLADAGFMKQYGECLGDVSARDLICAQKFNGLTVLPPLLTDEMIA
Rs87        PTKRSFIEDLLFNKVTLADAGFMKQYGECLGDVSARDLICAQKFNGLTVLPPLLTDEMIA
Rs68        PTKRSFIEDLLFNKVTLADAGFMKQYGECLGDVSARDLICAQKFNGLTVLPPLLTDEMIA
RaCH025     PTKRSFIEDLLFNKVTLADAGFMKQYGECLGDVSARDLICAQKFNGLTVLPPLLTDEMIA
RaCH039     PTKRSFIEDLLFNKVTLADAGFMKQYGECLGDVSARDLICAQKFNGLTVLPPLLTDEMIA
RaCH027     PTKRSFIEDLLFNKVTLADAGFMKQYGECLGDVSARDLICAQKFNGLTVLPPLLTDEMIA
Rs67        PTKRSFIEDLLFNKVTLADAGFMKQYGECLGDVSARDLICAQKFNGLTVLPPLLTDEMIA
Rs200609    PTKRSFIEDLLFNKVTLADAGFMKQYGECLGDVSARDLICAQKFNGLTVLPPLLTDEMIA
            ************************************************************

Rs56        AYTAALVSGTATAGWTFGAGAALQIPFAMQMAYRFNGIGVTQNVLYENQKLIANQFNSAI
Rs87        AYTAALVSGTATAGWTFGAGAALQIPFAMQMAYRFNGIGVTQNVLYENQKLIANQFNSAI
Rs68        AYTAALVSGTATAGWTFGAGAALQIPFAMQMAYRFNGIGVTQNVLYENQKLIANQFNSAI
RaCH025     AYTAALVSGTATAGWTFGAGAALQIPFAMQMAYRFNGIGVTQNVLYENQKLIANQFNSAI
RaCH039     AYTAALVSGTATAGWTFGAGAALQIPFAMQMAYRFNGIGVTQNVLYENQKLIANQFNSAI
RaCH027     AYTAALVSGTATAGWTFGAGAALQIPFAMQMAYRFNGIGVTQNVLYENQKLIANQFNSAI
Rs67        AYTAALVSGTATAGWTFGAGAALQIPFAMQMAYRFNGIGVTQNVLYENQKLIANQFNSAI
Rs200609    AYTAALVSGTATAGWTFGAGAALQIPFAMQMAYRFNGIGVTQNVLYENQKLIANQFNSAI
            ************************************************************

Rs56        GKIQESLSSTASALGKLQDVVNQNAQALNTLVKQLSSNFGAISSVLNDILSRLDKVEAEV
Rs87        GKIQESLSSTASALGKLQDVVNQNAQALNTLVKQLSSNFGAISSVLNDILSRLDKVEAEV
Rs68        GKIQESLSSTASALGKLQDVVNQNAQALNTLVKQLSSNFGAISSVLNDILSRLDKVEAEV
RaCH025     GKIQESLSSTASALGKLQDVVNQNAQALNTLVKQLSSNFGAISSVLNDILSRLDKVEAEV
RaCH039     GKIQESLSSTASALGKLQDVVNQNAQALNTLVKQLSSNFGAISSVLNDILSRLDKVEAEV
RaCH027     GKIQESLSSTASALGKLQDVVNQNAQALNTLVKQLSSNFGAISSVLNDILSRLDKVEAEV
Rs67        GKIQESLSSTASALGKLQDVVNQNAQALNTLVKQLSSNFGAISSVLNDILSRLDKVEAEV
Rs200609    GKIQESLSSTASALGKLQDVVNQNAQALNTLVKQLSSNFGAISSVLNDILSRLDKVEAEV
            ************************************************************

Rs56        QIDRLITGRLQSLQTYVTQQLIRAAEIRASANLAATKMSECVLGQSKRVDFCGKGYHLMS
Rs87        QIDRLITGRLQSLQTYVTQQLIRAAEIRASANLAATKMSECVLGQSKRVDFCGKGYHLMS
Rs68        QIDRLITGRLQSLQTYVTQQLIRAAEIRASANLAATKMSECVLGQSKRVDFCGKGYHLMS
RaCH025     QIDRLITGRLQSLQTYVTQQLIRAAEIRASANLAATKMSECVLGQSKRVDFCGKGYHLMS
RaCH039     QIDRLITGRLQSLQTYVTQQLIRAAEIRASANLAATKMSECVLGQSKRVDFCGKGYHLMS
RaCH027     QIDRLITGRLQSLQTYVTQQLIRAAEIRASANLAATKMSECVLGQSKRVDFCGKGYHLMS
Rs67        QIDRLITGRLQSLQTYVTQQLIRAAEIRASANLAATKMSECVLGQSKRVDFCGKGYHLMS
Rs200609    QIDRLITGRLQSLQTYVTQQLIRAAEIRASANLAATKMSECVLGQSKRVDFCGKGYHLMS
            ************************************************************

Rs56        FPQAAPHGVVFLHVTYVPSQEKNFTTAPAICHEGKAYFPREGVFVSNGTSWFITQRNFYS
Rs87        FPQAAPHGVVFLHVTYVPSQEKNFTTAPAICHEGKAYFPREGVFVSNGTSWFITQRNFYS
Rs68        FPQAAPHGVVFLHVTYVPSQEKNFTTAPAICHEGKAYFPREGVFVSNGTSWFITQRNFYS
```

```
RaCH025   FPQAAPHGVVFLHVTYVPSQEKNFTTAPAICHEGKAYFPREGVFVSNGTSWFITQRNFYS
RaCH039   FPQAAPHGVVFLHVTYVPSQEKNFTTAPAICHEGKAYFPREGVFVSNGTSWFITQRNFYS
RaCH027   FPQAAPHGVVFLHVTYVPSQEKNFTTAPAICHEGKAYFPREGVFVSNGTSWFITQRNFYS
Rs67      FPQAAPHGVVFLHVTYVPSQEKNFTTAPAICHEGKAYFPREGVFVSNGTSWFITQRNFYS
Rs200609  FPQSAPHGVVFLHVTYVPSQEKNFTTAPAICHEGKAYFPREGVFVSNGTSWFITQRNFYS
          ***:********************************************************

Rs56      PQLITTDNTFVSGNCDVVIGIINNTVYDPLQPELDSFKEELDKYFKNHTSPDVDLGDISG
Rs87      PQLITTDNTFVSGNCDVVIGIINNTVYDPLQPELDSFKEELDKYFKNHTSPDVDLGDISG
Rs68      PQLITTDNTFVSGNCDVVIGIINNTVYDPLQPELDSFKEELDKYFKNHTSPDVDLGDISG
RaCH025   PQLITTDNTFVSGNCDVVIGIINNTVYDPLQPELDSFKEELDKYFKNHTSPDVDLGDISG
RaCH039   PQLITTDNTFVSGNCDVVIGIINNTVYDPLQPELDSFKEELDKYFKNHTSPDVDLGDISG
RaCH027   PQLITTDNTFVSGNCDVVIGIINNTVYDPLQPELDSFKEELDKYFKNHTSPDVDLGDISG
Rs67      PQLITTDNTFVSGNCDVVIGIINNTVYDPLQPELDSFKEELDKYFKNHTSPDVDLGDISG
Rs200609  PQLITTDNTFVSGNCDVVIGIINNTVYDPLQPELDSFKDELDKYFKNHTSPDVDLGDISG
          **************************************:*********************

Rs56      INASVVNIQKDIDRLNEVAKNLNESLIDLQELGKYEQYIKWPWYVWLGFIAGLIAIVMVT
Rs87      INASVVNIQKDIDRLNEVAKNLNESLIDLQELGKYEQYIKWPWYVWLGFIAGLIAIVMVT
Rs68      INASVVNIQKEIDRLNEVAKNLNESLIDLQELGKYEQYIKWPWYVWLGFIAGLIAIVMVT
RaCH025   INASVVNIQKEIDRLNEVAKNLNESLIDLQELGKYEQYIKWPWYVWLGFIAGLIAIVMVT
RaCH039   INASVVNIQKEIDRLNEVAKNLNESLIDLQELGKYEQYIKWPWYVWLGFIAGLIAIVMVT
RaCH027   INASVVNIQKEIDRLNEVAKNLNESLIDLQELGKYEQYIKWPWYVWLGFIAGLIAIVMVT
Rs67      INASVVNIQKEIDRLNEVAKNLNESLIDLQELGKYEQYIKWPWYVWLGFIAGLIAIVMVT
Rs200609  INASVVNIQKEIDRLNEVAKNLNESLIDLQELGKYEQYIKWPWYVWLGFIAGLIAIVMVT
          **********:*************************************************

Rs56      ILLCCMTSCCSCLKGACSCGSCCKFDEDDSEPVLKGVKLHYT
Rs87      ILLCCMTSCCSCLKGACSCGSCCKFDEDDSEPVLKGVKLHYT
Rs68      ILLCCMTSCCSCLKGACSCGSCCKFDEDDSEPVLKGVKLHYT
RaCH025   ILLCCMTSCCSCLKGACSCGSCCKFDEDDSEPVLKGVKLHYT
RaCH039   ILLCCMTSCCSCLKGACSCGSCCKFDEDDSEPVLKGVKLHYT
RaCH027   ILLCCMTSCCSCLKGACSCGSCCKFDEDDSEPVLKGVKLHYT
Rs67      ILLCCMTSCCSCLKGACSCGSCCKFDEDDSEPVLKGVKLHYT
Rs200609  ILLCCMTSCCSCLKGACSCGSCCKFDEDDSEPVLKGVKLHYT
          ******************************************
```

Fig. S3 Amino acid sequence comparison of S gene of SARSr-CoV from Guangzhou and Huizhou, Guangdong Province
