# Supplementary figures and images for: Epidemiology and Genomic Characterization of Two Novel SARS-Related Coronaviruses in Horseshoe Bats from Guangdong, China
Source: mBio. 2022 Apr 25;13(3):e00463-22. doi: 10.1128/mbio.00463-22 (PMC9239062; doi:10.1128/mbio.00463-22)

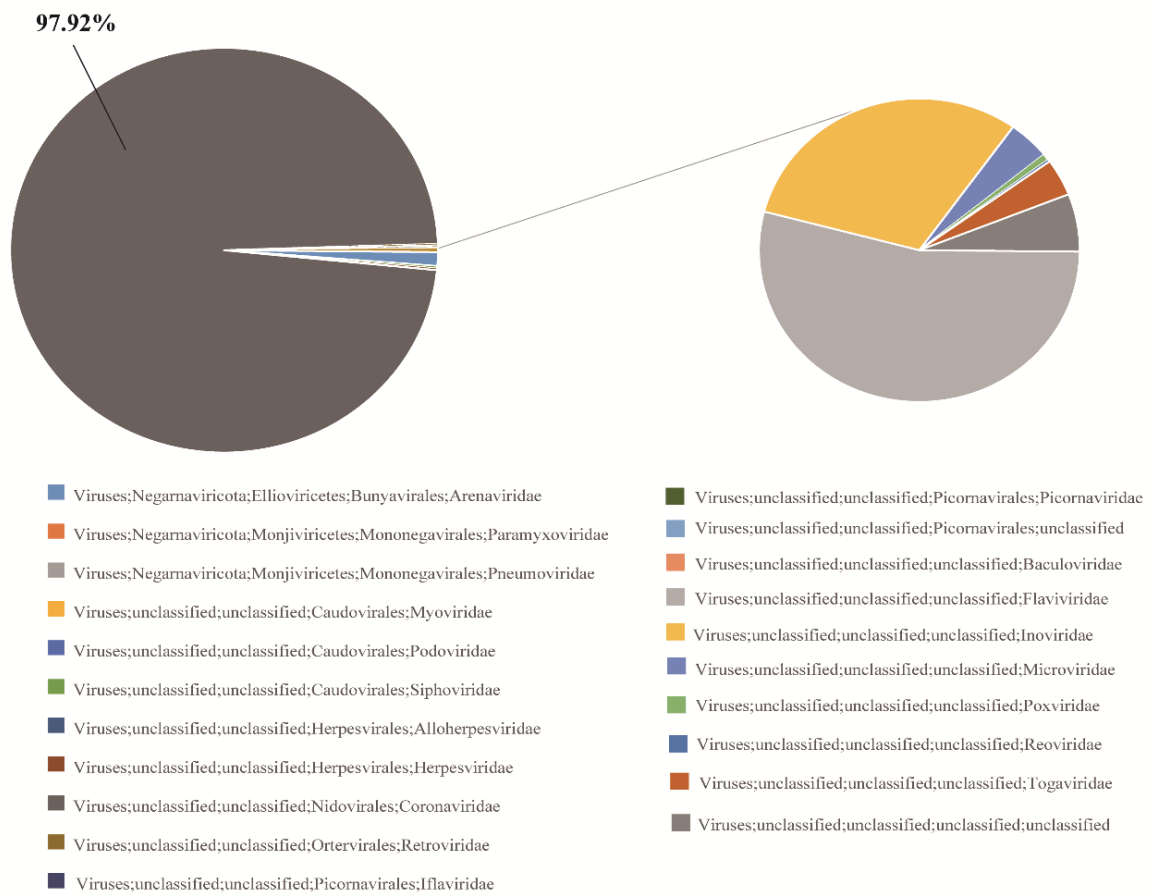

**Fig. S4** Virus reads classify at the family level by viral Metagenomics

Supplement: FIG S4 [file mbio.00463-22-sf004.pdf]
