# Supplementary material for: Epidemiology and Genomic Characterization of Two Novel SARS-Related Coronaviruses in Horseshoe Bats from Guangdong, China
Source: mBio. 2022 Apr 25;13(3):e00463-22. doi: 10.1128/mbio.00463-22 (PMC9239062; doi:10.1128/mbio.00463-22)
Supplement: TABLE S2 [file mbio.00463-22-st002.pdf]

**Table S2** Primers used to obtain the complete sequence and S gene of Bat SARSr-CoV. (A) Primers used to obtain the complete sequence of Bat SARSr-CoV-Rs56. (B) Primers used to obtain the complete sequence of Bat SARSr-CoV-RaCH025. (C) Primers used to obtain the S gene of CoV from positive samples.

**(A) Primers used to obtain the complete sequence of Bat SARSr-CoV-Rs56**

| Number | Primer | Sequence(5'-3') | Tm(℃) | Size(nt) | Location | Reference |
|---|---|---|---|---|---|---|
| 1 | 1F | ACCAACCAACTTTCGATCTCTTGT | 55 | 381 | 256-636 | ARTIC Network |
| | 1R | CATCTTTAAGATGTTGACGTGCCTC | | | | |
| 2 | 2F | CTGTTTTACAGGTTCGCGACGT | 55 | 385 | 568-952 | ARTIC Network |
| | 2R | TAAGGATCAGTGCCAAGCTCGT | | | | |
| 3 | 3F | CGGTAATAAAGGAGCTGGTGGC | 55 | 324 | 931-1254 | ARTIC Network |
| | 3R | AAGGTGTCTGCAATTCATAGCTCT | | | | |
| 4 | ZH22F | CGTGAGCACGAACATGAAGT | 55 | 501 | 1185-1685 | In this study |
| | ZH22R | GCGGAGTCGAGTTTCAATGT | | | | |
| 5 | ZH23F | TGTGAACAATGTGGCACTGA | 55 | 640 | 1512-2151 | In this study |
| | ZH23R | CATCCAGTGTGCGAGAGAAA | | | | |
| 6 | ZH3F | ACTGTGTGGTTTCCCCTCAC | 55 | 612 | 2087-2698 | In this study |
| | ZH3R | GGTGCCTTAAGAGGCATGAG | | | | |
| 7 | ZH4F | TTCATTGCACAAAGCAGAGG | 55 | 614 | 2616-3229 | In this study |
| | ZH4R | CCAGCGTCGTCAAACAAGTA | | | | |
| 8 | ZH5F | CCCCATGGGTATTGATCTTG | 55 | 640 | 3167-3806 | In this study |
| | ZH5R | AACATCCTCACCGGCATTTA | | | | |
| 9 | ZH6F | CTTGAAACATGGTGGTGGTG | 55 | 720 | 3614-4333 | In this study |
| | ZH6R | CCCGCCTTCTTAGCAGGTAT | | | | |
| 10 | ZH7F | AGGCATGTATCGACGAGGTC | 55 | 734 | 4111-4844 | In this study |
| | ZH7R | AACTGACACCACAGCAGGTG | | | | |
| 11 | 16F | AATTTGGAAGAAGCTGCTCGGT | 55 | 384 | 4785-5168 | ARTIC Network |
| | 16R | CACAACTTGCGTGTGGAGGTTA | | | | |
| 12 | ZH8F | TTCTTAAGCGTGGGGACAAG | 55 | 798 | 4993-5790 | In this study |
| | ZH8R | TACGCCCACATACACAAGGA | | | | |
| 13 | ZH9F | GCCAGAAAACAACCACCTTG | 55 | 629 | 5683-6311 | In this study |
| | ZH9R | GCCATTCAAGTCTGGGAAGA | | | | |
| 14 | ZH9-12F | GCCATTGCCAAATGCGAGTT | 55 | 1382 | 6170-7551 | In this study |
| | ZH9-12R | CAGAAACGGGTGCCATTTGT | | | | |
| 15 | ZH56C2F1 | TGTTAGGGGTGTTGCTAGGT | 55 | 530 | 7022-7551 | In this study |
| | ZH9-12R | CAGAAACGGGTGCCATTTGT | | | | |
| 16 | ZH12F | CCTGACATTTTTGGGTTTGG | 55 | 763 | 7367-8129 | In this study |
| | ZH12R | TAACAGAATGGGTTGGCACA | | | | |
| 17 | ZH30F | ATGGCGCACTTCACCTCTAC | 55 | 1030 | 7909-8938 | In this study |
| | ZH30R | CGCTGGCTAAACCATGAGTC | | | | |
| 18 | ZH14F | ATGTTTCGCTGGTTTGGAAC | 55 | 677 | 8539-9215 | In this study |
| | ZH14R | ACAGTATGGCACCGGCTTAC | | | | |
| 19 | J1F | TGCCTGGTTTACCTGGTACTG | 55 | 397 | | |

| | | | | | | |
|---|---|---|---|---|---|---|
| | J1R | AAGCACACACCAGCTTCTGA | | | 9013-9409 | In this study |
| 20 | ZH15F | GTAAGCCGGTGCCATACTGT | 55 | 604 | 9196- | In this |
| | ZH15R | CATTGCAGATGAGCCAAGAA | | | 9799 | study |
| 21 | J2F | ACTCTGTCTGGCACCTGCTT | 55 | 843 | 9689- | In this |
| | J2R | ACCTGGTTGGATACGGACAA | | | 10531 | study |
| 22 | J3F | ACCCGAACTATGACGACCTG | 55 | 905 | 10357- | In this |
| | J3R | AGCAAATGGCAAGAAAGCAT | | | 11261 | study |
| 23 | J4F | TGTTCTGGTGTGACCTTCCA | 55 | 1048 | 11103- | In this |
| | J4R | CCATCTTTTCGAAAGCTTCA | | | 12150 | study |
| 24 | 40F | TGCACATCAGTAGTCTTACTCTCAGT | 55 | 393 | 12015- | ARTIC |
| | 40R | CATGGCTGCATCACGGTCAAAT | | | 12407 | Network |
| 25 | J5F | AGGCCTATGAGCAGGCTGTA | 55 | 278 | 12301- | In this |
| | J5R | ATCACGCGCATTGTTGATAA | | | 12578 | study |
| 26 | ZH24F | TCTGAGTTTGACCGTGATGC | 55 | 908 | 12381- | In this |
| | ZH24R | ATTGGTTGTCCTCCACTTGC | | | 13288 | study |
| 27 | 44F | TGCCACAGTACGTCTACAAGCT | 55 | 395 | 13157- | ARTIC |
| | 44R | AACCTTTCCACATACCGCAGAC | | | 13551 | Network |
| 28 | 45F | AGTATGTACAAATACCTACAACTTGTGCT | 55 | 392 | 13459- | ARTIC |
| | 45R | AAATTGTTTCTTCATGTTGGTAGTTAGAGA | | | 13850 | Network |
| 29 | 45F | TACCTACAACTTGTGCTAATGACCC | 55 | 1607 | 13471- | ARTIC |
| | 49R | TGACGATGACTTGGTTAGCATTAATACA | | | 15077 | Network |
| 30 | 49F | AGGAATTACTTGTGTATGCTGCTGA | 55 | 381 | 14697- | ARTIC |
| | 49R | TGACGATGACTTGGTTAGCATTAATACA | | | 15077 | Network |
| 31 | 49F | AGGAATTACTTGTGTATGCTGCTGA | 55 | 1341 | 14697- | ARTIC |
| | 52R | GTTGAGAGCAAAATTCATGAGGTCC | | | 16037 | Network |
| 32 | 51F | TCAATAGCCGCCACTAGAGGAG | 55 | 715 | 15323- | ARTIC |
| | 52R | GTTGAGAGCAAAATTCATGAGGTCC | | | 16037 | Network |
| 33 | 52F | CATCAGGAGATGCCACAACTGC | 55 | 405 | 15633- | ARTIC |
| | 52R | GTTGAGAGCAAAATTCATGAGGTCC | | | 16037 | Network |
| 34 | 53F | AGCAAAATGTTGGACTGAGACTGA | 55 | 382 | 15979- | ARTIC |
| | 53R | AGCCTCATAAAACTCAGGTTCCC | | | 16360 | Network |
| 35 | 53F | AGCAAAATGTTGGACTGAGACTGA | 55 | 1006 | 15979- | ARTIC |
| | 55R | GGTGTACTCTCCTATTTGTACTTTACTGT | | | 16984 | Network |
| 36 | 55F | ACTCAACTTTACTTAGGAGGTATGAGCT | 55 | 417 | 16568- | ARTIC |
| | 55R | GGTGTACTCTCCTATTTGTACTTTACTGT | | | 16984 | Network |
| 37 | 56F | ACCTAGACCACCACTTAACCGA | 55 | 404 | 16900- | ARTIC |
| | 56R | ACACTATGCGAGCAGAAGGGTA | | | 17303 | Network |
| 38 | 57F | ATTCTACACTCCAGGGACCACC | 55 | 387 | 17217- | ARTIC |
| | 57R | GTAATTGAGCAGGGTCGCCAAT | | | 17603 | Network |
| 39 | 58F | TGATTTGAGTGTTGTCAATGCCAGA | 55 | 380 | 17533- | ARTIC |
| | 58R | CTTTTCTCCAAGCAGGGTTACGT | | | 17912 | Network |
| 40 | 59F | TCACGCATGATGTTTCATCTGCA | 55 | 388 | 17826- | ARTIC |
| | 59R | AAGAGTCCTGTTACATTTTCAGCTTG | | | 18213 | Network |

| | | | | | | |
|---|---|---|---|---|---|---|
| 41 | 60F<br>60R | TGATAGAGACCTTTATGACAAGTTGCA<br>GGTACCAACAGCTTCTCTAGTAGC | 55 | 380 | 18120-<br>18499 | ARTIC<br>Network |
| 42 | ZH60-63F<br>ZH60-63R | GCTATTCGTCACGTTCGTGC<br>CAAGGCTGAGCGTCGTAGAA | 55 | 833 | 18425-<br>19257 | In this<br>study |
| 43 | 63F<br>63R | TGTTAAGCGTGTTGACTGGACT<br>ACAAACTGCCACCATCACAACC | 55 | 401 | 19048-<br>19448 | ARTIC<br>Network |
| 44 | J6F<br>J6R | GCAGCGTGCAGAAAAGTACA<br>AAAGCTGGAGTGTGGAATGC | 55 | 374 | 19109-<br>19482 | In this<br>study |
| 45 | ZH17F<br>ZH17R | ATCGTTACCCAGCCAATGCA<br>TCTCCATTTGTGACCTGGGC | 55 | 1044 | 19359-<br>20402 | In this<br>study |
| 46 | J7F<br>J7R | AATGCCCGTAATGGTGTTTT<br>CCATGCCTGACTTGCTTGTA | 55 | 648 | 20177-<br>20824 | In this<br>study |
| 47 | 68F<br>68R | ACAGGTTCATCTAAGTGTGTGTGT<br>CTCCTTTATCAGAACCAGCACCA | 55 | 417 | 20624-<br>21041 | ARTIC<br>Network |
| 48 | J8F<br>J8R | TGCTTCTTGAAAAATGTGACCT<br>GGGTTTGTGTTCCTCCAAAA | 55 | 648 | 20868-<br>21516 | In this<br>study |
| 49 | ZH19F<br>ZH19R | TCATGGTGGACAGCCTTTGT<br>GCATCCGTGATCGTCCCATT | 55 | 1177 | 21371-<br>22548 | In this<br>study |
| 50 | ZHS2F<br>ZHS2R | GAGGCTGGATTTTTGGTTCA<br>ATTCAGCAAGTGGGTTTTGG | 55 | 555 | 22026-<br>22580 | In this<br>study |
| 51 | J9F<br>J9R | CCTAGAGGTTTGCCTGAAGGT<br>GAGAAATTGAAACCGCCAAA | 55 | 1685 | 22340-<br>24024 | In this<br>study |
| 52 | ZH21F<br>ZH21R | GGAACGAACGCCTCATCAGA<br>ACCTGGGCAAAGACTTCCTG | 55 | 542 | 23429-<br>23970 | In this<br>study |
| 53 | ZH2F<br>ZH2R | TGGAAGCTTTTGCACACAAC<br>GCAGCTGTGTATGCAGCAAT | 55 | 360 | 23884-<br>24243 | In this<br>study |
| 54 | 80F<br>80R | TTGCCTTGGTGATATTGCTGCT<br>TGGAGCTAAGTTGTTTAACAAGCG | 55 | 389 | 24133-<br>24521 | ARTIC<br>Network |
| 55 | ZH1F<br>ZH1R | AGGAAAATTGCAGGATGTGG<br>GCGGTCAATCTCCTTTTGAA | 55 | 721 | 24451-<br>25171 | In this<br>study |
| 56 | 83F<br>83R | TCCTTTGCAACCTGAATTAGACTCA<br>TTTGACTCCTTTGAGCACTGGC | 55 | 395 | 25029-<br>25423 | ARTIC<br>Network |
| 57 | ZH26F<br>ZH26R | ATGACCCTCTGCAACCTGAG<br>ACACCTGAATGCCAATCCTC | 55 | 990 | 25048-<br>26037 | In this<br>study |
| 58 | J10F<br>J10R | ATCATGAGATGCTGGCTGTG<br>CCCCAGTCTCAGTCGACAAT | 55 | 304 | 25817-<br>26120 | In this<br>study |
| 59 | 86F<br>86R | TCAGGTGATGGCACAACAAGTC<br>ACGAAAGCAAGAAAAAGAAGTACGC | 55 | 410 | 25958-<br>26367 | ARTIC<br>Network |
| 60 | 87F<br>89R | CGACTACTAGCGTGCCTTTGTA<br>ACCTGAAAGTCAACGAGATGAAACA | 55 | 1030 | 26250-<br>27279 | ARTIC<br>Network |
| 61 | 87F<br>87R | CGACTACTAGCGTGCCTTTGTA<br>ACTAGGTTCCATTGTTCAAGGAGC | 55 | 372 | 26271-<br>26642 | ARTIC<br>Network |
| 62 | 89F<br>89R | GTACGCGTTCCATGTGGTCATT<br>ACCTGAAAGTCAACGAGATGAAACA | 55 | 392 | 26888-<br>27279 | ARTIC<br>Network |

| | | | | | | |
|---|---|---|---|---|---|---|
| 63 | 89F | GTACGCGTTCCATGTGGTCATT | 55 | 1029 | 26888- | ARTIC |
| | 91R | TTCAAGTGAGAACCAAAAGATAATAAGCA | | | 27916 | Network |
| 64 | 91F | TCACTACCAAGAGTGTGTTAGAGGT | 55 | 411 | 27506- | ARTIC |
| | 91R | TTCAAGTGAGAACCAAAAGATAATAAGCA | | | 27916 | Network |
| 65 | 91F | TCACTACCAAGAGTGTGTTAGAGGT | 55 | 1329 | 27506- | ARTIC |
| | 94R | TTTGGCAATGTTGTTCCTTGAGG | | | 28834 | Network |
| 66 | 94F | GGCCCCAAGGTTTACCCAATAA | 55 | 385 | 28450- | ARTIC |
| | 94R | TTTGGCAATGTTGTTCCTTGAGG | | | 28834 | Network |
| 67 | 95F | TGAGGGAGCCTTGAATACACCA | 55 | 386 | 28733- | ARTIC |
| | 95R | CAGTACGTTTTTGCCGAGGCTT | | | 29118 | Network |
| 68 | 95F | TGAGGGAGCCTTGAATACACCA | 55 | 1024 | 28733- | ARTIC |
| | 97R | ACACACTGATTAAAGATTGCTATGTGAG | | | 29756 | Network |
| 69 | 97F | TGGATGACAAAGATCCAAATTTCAAAGA | 55 | 413 | 29344- | ARTIC |
| | 97R | ACACACTGATTAAAGATTGCTATGTGAG | | | 29756 | Network |
| 70 | 97F | TGGATGACAAAGATCCAAATTTCAAAGA | 55 | 586 | 29344- | ARTIC |
| | 98R | TTCTCCTAAGAAGCTATTAAAATCACATGG | | | 29929 | Network |

**(B) Primers used to obtain the complete sequence of Bat SARSr-CoV-RaCH025**

| Number | Primer | Sequence(5'-3') | Tm(℃) | Size(nt) | Location | Reference |
|---|---|---|---|---|---|---|
| 1 | 1F | ACCAACCAACTTTCGATCTCTTGT | 55 | 941 | 1-941 | ARTIC |
| | 3R | AAGGTGTCTGCAATTCATAGCTCT | | | | Network |
| 2 | 3F | CGGTAATAAAGGAGCTGGTGGC | 55 | 817 | 556-1372 | ARTIC Network |
| | ZH22R | GCGGAGTCGAGTTTCAATGT | | | | In this study |
| 3 | ZH22F | CGTGAGCACGAACATGAAGT | 55 | 501 | 872-1372 | In this study |
| | ZH22R | GCGGAGTCGAGTTTCAATGT | | | | |
| 4 | ZH23F | TGTGAACAATGTGGCACTGA | 55 | 640 | 1199- | In this study |
| | ZH23R | CATCCAGTGTGCGAGAGAAA | | | 1838 | |
| 5 | ZH3F | ACTGTGTGGTTTCCCCTCAC | 55 | 612 | 1774- | In this study |
| | ZH3R | GGTGCCTTAAGAGGCATGAG | | | 2385 | |
| 6 | ZH4F | TTCATTGCACAAAGCAGAGG | 55 | 614 | 2303- | In this study |
| | ZH4R | CCAGCGTCGTCAAACAAGTA | | | 2916 | |
| 7 | ZH5F | CCCCATGGGTATTGATCTTG | 55 | 640 | 2854- | In this study |
| | ZH5R | AACATCCTCACCGGCATTTA | | | 3493 | |
| 8 | ZH6F | CTTGAAACATGGTGGTGGTG | 55 | 720 | 3301- | In this study |
| | ZH6R | CCCGCCTTCTTAGCAGGTAT | | | 4020 | |
| 9 | ZH7F | AGGCATGTATCGACGAGGTC | 55 | 734 | 3798- | In this study |
| | ZH7R | AACTGACACCACAGCAGGTG | | | 4531 | |
| 10 | ZH7F | AGGCATGTATCGACGAGGTC | 53 | 1058 | 3798- | ARTIC Network |
| | 16R | CACAACTTGCGTGTGGAGGTTA | | | 4855 | In this study |
| 11 | ZH8F | TTCTTAAGCGTGGGGACAAG | 55 | 798 | 4680- | In this study |
| | ZH8R | TACGCCCACATACACAAGGA | | | 5477 | |
| 12 | ZH9F | GCCAGAAAACAACCACCTTG | 55 | 629 | 5370- | In this study |
| | ZH9R | GCCATTCAAGTCTGGGAAGA | | | 5998 | |
| 13 | ZH9-12F | GCCATTGCCAAATGCGAGTT | 55 | 1382 | | In this study |

| | | | | | | |
|---|---|---|---|---|---|---|
| | ZH9-12R | CAGAAACGGGTGCCATTTGT | | | 5857-7238 | |
| 14 | ZH12F<br>ZH12R | CCTGACATTTTTGGGTTTGG<br>TAACAGAATGGGTTGGCACA | 55 | 763 | 7054-7816 | In this study |
| 15 | ZH30F<br>ZH30R | ATGGCGCACTTCACCTCTAC<br>CGCTGGCTAAACCATGAGTC | 55 | 1030 | 7596-8625 | In this study |
| 16 | ZH30F<br>J1R | ATGGCGCACTTCACCTCTAC<br>AAGCACACACCAGCTTCTGA | 53 | 1501 | 7596-9096 | In this study |
| 17 | J1F<br>J1R | TGCCTGGTTTACCTGGTACTG<br>AAGCACACACCAGCTTCTGA | 55 | 397 | 8700-9096 | In this study |
| 18 | J1F<br>J2R | TGCCTGGTTTACCTGGTACTG<br>ACCTGGTTGGATACGGACAA | 53 | 1520 | 8700-10219 | In this study |
| 19 | J2F<br>J2R | ACTCTGTCTGGCACCTGCTT<br>ACCTGGTTGGATACGGACAA | 55 | 844 | 9376-10219 | In this study |
| 20 | J2F<br>35R | ACTCTGTCTGGCACCTGCTT<br>ACTTCATAGCCACAAGGTTAAAGTCA | 55 | 1226 | 9376-10601 | In this study<br>ARTIC Network |
| 21 | 35F<br>35R | TGTTCGCATTCAACCAGGACAG<br>ACTTCATAGCCACAAGGTTAAAGTCA | 55 | 402 | 10200-10601 | ARTIC Network |
| 22 | 36F<br>36R | TTAGCTTGGTTGTACGCTGCTG<br>GAACAAAGACCATTGAGTACTCTGGA | 55 | 408 | 10505-10912 | ARTIC Network |
| 23 | J4F<br>J4R | TGTTCTGGTGTGACCTTCCA<br>CCATCTTTTCGAAAGCTTCA | 55 | 1048 | 10790-11837 | In this study |
| 24 | 40F<br>40R | TGCACATCAGTAGTCTTACTCTCAGT<br>CATGGCTGCATCACGGTCAAAT | 55 | 393 | 11702-12094 | ARTIC Network |
| 25 | J5F<br>J5R | AGGCCTATGAGCAGGCTGTA<br>ATCACGCGCATTGTTGATAA | 55 | 278 | 11988-12265 | In this study |
| 26 | ZH24F<br>ZH24R | TCTGAGTTTGACCGTGATGC<br>ATTGGTTGTCCTCCACTTGC | 55 | 908 | 12068-12975 | In this study |
| 27 | ZH24F<br>45R | TCTGAGTTTGACCGTGATGC<br>AAATTGTTTCTTCATGTTGGTAGTTAGAGA | 53 | 1470 | 12068-13537 | In this study<br>ARTIC Network |
| 28 | 45F<br>45R | AGTATGTACAAATACCTACAACTTGTGCT<br>AAATTGTTTCTTCATGTTGGTAGTTAGAGA | 55 | 392 | 13146-13537 | ARTIC Network |
| 29 | 45F<br>49R | AGTATGTACAAATACCTACAACTTGTGCT<br>TGACGATGACTTGGTTAGCATTAATACA | 55 | 1619 | 13146-14764 | ARTIC Network |
| 30 | 49F<br>52R | AGGAATTACTTGTGTATGCTGCTGA<br>GTTGAGAGCAAAATTCATGAGGTCC | 55 | 1341 | 14384-15724 | ARTIC Network |
| 31 | 52F<br>52R | CATCAGGAGATGCCACAACTGC<br>GTTGAGAGCAAAATTCATGAGGTCC | 55 | 405 | 15320-15724 | ARTIC Network |
| 32 | 53F<br>53R | AGCAAAATGTTGGACTGAGACTGA<br>AGCCTCATAAAACTCAGGTTCCC | 55 | 382 | 15666-16047 | ARTIC Network |
| 33 | 53F<br>55R | AGCAAAATGTTGGACTGAGACTGA<br>GGTGTACTCTCCTATTTGTACTTTACTGT | 55 | 1006 | 15666-16671 | ARTIC Network |
| 34 | 55F<br>55R | ACTCAACTTTACTTAGGAGGTATGAGCT<br>GGTGTACTCTCCTATTTGTACTTTACTGT | 55 | 417 | 16255-16671 | ARTIC Network |

| | | | | | | |
|---|---|---|---|---|---|---|
| 35 | 55F<br>56R | ACTCAACTTTACTTAGGAGGTATGAGCT<br>ACACTATGCGAGCAGAAGGGTA | 53 | 736 | 16255-<br>16990 | ARTIC<br>Network |
| 36 | 57F<br>57R | ATTCTACACTCCAGGGACCACC<br>GTAATTGAGCAGGGTCGCCAAT | 55 | 387 | 16904-<br>17290 | ARTIC<br>Network |
| 37 | 58F<br>58R | TGATTTGAGTGTTGTCAATGCCAGA<br>CTTTTCTCCAAGCAGGGTTACGT | 55 | 380 | 17220-<br>17599 | ARTIC<br>Network |
| 38 | 59F<br>59R | TCACGCATGATGTTTCATCTGCA<br>AAGAGTCCTGTTACATTTTCAGCTTG | 55 | 388 | 17513-<br>17900 | ARTIC<br>Network |
| 39 | 60F<br>60R | TGATAGAGACCTTTATGACAAGTTGCA<br>GGTACCAACAGCTTCTCTAGTAGC | 55 | 382 | 17805-<br>18186 | ARTIC<br>Network |
| 40 | ZH60-63F<br>ZH60-63R | GCTATTCGTCACGTTCGTGC<br>CAAGGCTGAGCGTCGTAGAA | 55 | 833 | 18112-<br>18944 | In this study |
| 41 | 63F<br>63R | TGTTAAGCGTGTTGACTGGACT<br>ACAAACTGCCACCATCACAACC | 55 | 401 | 18735-<br>19135 | ARTIC<br>Network |
| 42 | J6F<br>J6R | GCAGCGTGCAGAAAAGTACA<br>AAAGCTGGAGTGTGGAATGC | 55 | 374 | 18796-<br>19169 | In this study |
| 43 | ZH17F<br>ZH17R | ATCGTTACCCAGCCAATGCA<br>TCTCCATTTGTGACCTGGGC | 55 | 1044 | 19046-<br>20089 | In this study |
| 44 | J7F<br>J7R | AATGCCCGTAATGGTGTTTT<br>CCATGCCTGACTTGCTTGTA | 55 | 648 | 19864-<br>20511 | In this study |
| 45 | 68F<br>68R | ACAGGTTCATCTAAGTGTGTGTGT<br>CTCCTTTATCAGAACCAGCACCA | 55 | 418 | 20311-<br>20728 | ARTIC<br>Network |
| 46 | J8F<br>J8R | TGCTTCTTGAAAAATGTGACCT<br>GGGTTTGTGTTCCTCCAAAA | 55 | 649 | 20555-<br>21203 | In this study |
| 47 | ZH19F<br>ZH19R | TCATGGTGGACAGCCTTTGT<br>GCATCCGTGATCGTCCCATT | 55 | 1178 | 21058-<br>22235 | In this study |
| 48 | J9F<br>J9R | CCTAGAGGTTTGCCTGAAGGT<br>GAGAAATTGAAACCGCCAAA | 55 | 1685 | 22027-<br>23711 | In this study |
| 49 | ZH2F<br>ZH2R | TGGAAGCTTTTGCACACAAC<br>GCAGCTGTGTATGCAGCAAT | 55 | 360 | 23571-<br>23930 | In this study |
| 50 | 80F<br>80R | TTGCCTTGGTGATATTGCTGCT<br>TGGAGCTAAGTTGTTTAACAAGCG | 55 | 389 | 23820-<br>24208 | ARTIC<br>Network |
| 51 | ZH1F<br>ZH1R | AGGAAAATTGCAGGATGTGG<br>GCGGTCAATCTCCTTTTGAA | 55 | 721 | 24138-<br>24858 | In this study |
| 52 | ZH26F<br>ZH26R | ATGACCCTCTGCAACCTGAG<br>ACACCTGAATGCCAATCCTC | 55 | 1009 | 24716-<br>25724 | In this study |
| 53 | J10F<br>J10R | ATCATGAGATGCTGGCTGTG<br>CCCCAGTCTCAGTCGACAAT | 55 | 304 | 25504-<br>25807 | In this study |
| 54 | 86F<br>86R | TCAGGTGATGGCACAACAAGTC<br>ACGAAAGCAAGAAAAAGAAGTACGC | 55 | 410 | 25645-<br>26054 | ARTIC<br>Network |
| 55 | 87F<br>89R | CGACTACTAGCGTGCCTTTGTA<br>ACCTGAAAGTCAACGAGATGAAACA | 55 | 1030 | 25937-<br>26966 | ARTIC<br>Network |
| 56 | 89F<br>89R | GTACGCGTTCCATGTGGTCATT<br>ACCTGAAAGTCAACGAGATGAAACA | 55 | 392 | 26575-<br>26966 | ARTIC<br>Network |

| | | | | | | |
|---|---|---|---|---|---|---|
| 57 | 89F | GTACGCGTTCCATGTGGTCATT | 55 | 704 | 26575- | ARTIC |
| | 90R | TGAAATGGTGAATTGCCCTCGT | | | 27278 | Network |
| 58 | 91F | TCACTACCAAGAGTGTGTTAGAGGT | 55 | 411 | 27192- | ARTIC |
| | 91R | TTCAAGTGAGAACCAAAAGATAATAAGCA | | | 27602 | Network |
| 59 | 91F | TCACTACCAAGAGTGTGTTAGAGGT | 55 | 1339 | 27192- | ARTIC |
| | 94R | TTTGGCAATGTTGTTCCTTGAGG | | | 28530 | Network |
| 60 | 94F | GGCCCCAAGGTTTACCCAATAA | 55 | 385 | 28146- | ARTIC |
| | 94R | TTTGGCAATGTTGTTCCTTGAGG | | | 28530 | Network |
| 61 | 95F | TGAGGGAGCCTTGAATACACCA | 55 | 386 | 28429- | ARTIC |
| | 95R | CAGTACGTTTTTGCCGAGGCTT | | | 28814 | Network |
| 62 | 96F | GCCAACAACAACAAGGCCAAAC | 55 | 393 | 28737- | ARTIC |
| | 96R | TAGGCTCTGTTGGTGGGAATGT | | | 29129 | Network |
| 63 | 97F | TGGATGACAAAGATCCAAATTTCAAAGA | 55 | 413 | 29040- | ARTIC |
| | 97R | ACACACTGATTAAAGATTGCTATGTGAG | | | 29452 | Network |
| 64 | 97F | TGGATGACAAAGATCCAAATTTCAAAGA | 55 | 583 | 29040- | ARTIC |
| | 98R | TTCTCCTAAGAAGCTATTAAAATCACATGG | | | 29622 | Network |

**(C) Primers used to obtain the S gene of CoV from positive samples**

| Number | primer | Sequence(5'-3') | Tm (℃) | Size(nt) | Template/ Sample number |
|---|---|---|---|---|---|
| 1 | ZH19F | TCATGGTGGACAGCCTTTGT | 55 | 1177 | 56，67，68，87 |
| | ZH19R | GCATCCGTGATCGTCCCATT | | | |
| 2 | ZHS2F | GAGGCTGGATTTTTGGTTCA | 55 | 555 | 56，67，68，87，200609，200634, 183 |
| | ZHS2R | ATTCAGCAAGTGGGTTTTGG | | | |
| 3 | J9F | CCTAGAGGTTTGCCTGAAGGT | 55 | 1685 | 56，67，68，87 |
| | J9R | GAGAAATTGAAACCGCCAAA | | | |
| 4 | ZH2F | TGGAAGCTTTTGCACACAAC | 55 | 360 | 56，67，68，87，52，200609, 200634, 150,183 |
| | ZH2R | GCAGCTGTGTATGCAGCAAT | | | |
| 5 | 80F | TTGCCTTGGTGATATTGCTGCT | 55 | 389 | 56，67，68，87，52，200609, 150 |
| | 80R | TGGAGCTAAGTTGTTTAACAAGCG | | | |
| 6 | ZH1F | AGGAAAATTGCAGGATGTGG | 55 | 721 | 56，67，68，87，52，200609 |
| | ZH1R | GCGGTCAATCTCCTTTTGAA | | | |
| 7 | ZH26F | ATGACCCTCTGCAACCTGAG | 55 | 1009 | 56，67，68，87，52 |
| | ZH26R | ACACCTGAATGCCAATCCTC | | | |
| 8 | 609S1F | GAGGGACGGCTGTTATGTCT | | | |
| | 609S1R | TGGCAGGAAGTAATCCTGTG | 55 | 324 | 52, 200609 |
| 9 | 609S6F | CCAGGCGAAACTGGTGTTAT | | | |
| | 609S6R | CCTCTGATGAGGCATTCGTT | 55 | 532 | 52, 200609, 150 |
| 10 | 609S7F | TTCACCTTGCTCATTTGGTG | | | |
| | 609S7R | AACCTGGGCAAAAACTTCCT | 55 | 583 | 52 |
| 11 | 609S8F | ATGACCCTCTGCAACCTGAG | | | |
| | 609S8R | TGATTGCTCCCAATGTGAAA | 55 | 459 | 52, 200609 |
| 12 | 609-3F | GTGACCCGCAGACCTTAGAAA | | | |
| | 609-3R | AGTGAGGGCACGATTGAGTT | 55 | 563 | 200609 |
