# Supplementary material for: Epidemiology and Genomic Characterization of Two Novel SARS-Related Coronaviruses in Horseshoe Bats from Guangdong, China
Source: mBio. 2022 Apr 25;13(3):e00463-22. doi: 10.1128/mbio.00463-22 (PMC9239062; doi:10.1128/mbio.00463-22)
Supplement: TABLE S3 [file mbio.00463-22-st003.pdf]

Table S3 Information of *Betacoronaviruses* in GenBank database

Number	Name	Host	GenBank number	Sampling date	Place of isolation
1	SARS-CoV-GZ02	Human	AY390556	11/02/2003	Guangdong
2	SARS-CoV-Tor2	Human	AY274119.3	03/2003	Toronto
3	SARS-CoV-BJ01	Human	AY278488.2	2003	Beijing
4	SARS-CoV-2	Human	MN908947.3	12/2019	Hubei
5	SARS-CoV-civet010	Civet	AY572035	02/01/2004	Guangdong
6	SARS-CoV-civet020	Civet	AY572038	02/01/2004	Guangdong
7	SARS-CoV-SZ3	Civet	AY304486	15/05/2003	Guangdong
8	Pangolin-CoV-2020	Pangolin	MT121216	03/29/2019	Guangdong
9	Bat-SARSr-CoV-Rs3367	*Rhinolophus sinicus*	KC881006	03/19/2012	Yunnan
10	Bat_SARSr_CoV_Rs9401	*Rhinolophus sinicus*	KY417152	10/16/2015	Yunnan
11	Bat_SARSr_CoV_Rs7327	*Rhinolophus sinicus*	KY417151	10/24/2014	Yunnan
12	Bat-SARSr-CoV-WIVI	*Rhinolophus sinicus*	KF367457	09/2012	Yunnan
13	Bat-SARSr-CoV-Rs4874	*Rhinolophus sinicus*	KY417150	07/21/2013	Yunnan
14	Bat-SARSr-CoV-YN2020B	*Rhinolophus sinicus*	OK017852	06/2020	Yunnan
15	Bat-SARSr-CoV-YN2020C	*Rhinolophus sinicus*	OK017853	06/2020	Yunnan
16	Bat-SARSr-CoV-YN2020D	*Rhinolophus sinicus*	OK017854	06/2020	Yunnan
17	Bat-SARSr-CoV-YN2020E	*Rhinolophus sinicus*	OK017855	06/2020	Yunnan
18	Bat-SARSr-CoV-Rs4255	*Rhinolophus sinicus*	KY417149	04/17/2013	Yunnan
19	Bat-SARSr-CoV-Rf4092	*Rhinilophus ferrumequinum*	KY417145	09/18/2012	Yunnan
20	Bat-SARSr-CoV-YN2013	*Rhinolophus sinicus*	KJ473816	2013	Yunnan
21	Bat_SARSr_CoV_YN2018D	*Rhinolophus affinis*	MK211378	09/2016	Yunnan
22	Bat-CoV-LYRa11	*Rhinolophus affinis*	KF569996	2011	Yunnan
23	Bat-CoV-RaTG13	*Rhinolophus affinis*	MN996532.2	07/24/2013	Yunnan
24	Bat-SARSr-CoV-Rs672	*Rhinolophus sinicus*	FJ588686	2006	Guizhou
25	Bat-SARSr-CoV-GZ2021I	*Rhinolophus sinicus*	OK017831	03/2021	Guizhou
26	Bat-SARSr-CoV-GZ2021C	*Rhinolophus sinicus*	OK017829	03/2021	Guizhou
27	Bat-SARSr-CoV-GZ2021H	*Rhinolophus sinicus*	OK017830	03/2021	Guizhou
28	Bat-SARSr-CoV-GX2013	*Rhinolophus sinicus*	KJ473815	2013	Guangxi
29	Bat-SARSr-CoV-Rp3	*Rhinolophus pearsoni*	DQ071615	2004/12/4	Guangxi
30	Bat-SARSr-CoV-GX2019A	*Rhinolophus siamensis*	OK017859	09/2019	Guangxi
31	Bat-SARSr-CoV-HuB2013	*Rhinolophus sinicus*	KJ473814	2013	Hubei
32	Bat-SARSr-CoV-279/04	*Rhinolophus ferrumequinum*	DQ648857	2004	Hubei
33	Bat-SARSr-CoV-273/04	*Rhinolophus macrotis*	DQ648856	2004	Hubei
34	Bat-SARSr-CoV-Rm1	*Rhinolophus macrotis*	DQ412043	2004/11/4	Hubei

35	Bat-SARSr-CoV-Rf1	*Rhinolophus ferrumequinum*	DQ412042	2004/11/4	Hubei
36	Bat-SARSr-CoV-LN2020A	*Rhinolophus ferrumequinum*	OK017794	09/2020	Liaoning
37	Bat-SARSr-CoV-LN2020B	*Rhinolophus ferrumequinum*	OK017795	09/2020	Liaoning
38	Bat-SARSr-CoV-LN2020C	*Rhinolophus ferrumequinum*	OK017796	09/2020	Liaoning
39	Bat-SARSr-CoV-LN2020E	*Rhinolophus ferrumequinum*	OK017797	09/2020	Liaoning
40	Bat-SARSr-CoV-LN2020F	*Rhinolophus ferrumequinum*	OK017798	09/2020	Liaoning
41	Bat-SARSr-CoV-LN2021G	*Rhinolophus ferrumequinum*	OK017799	09/2020	Liaoning
42	Bat-SARSr-CoV-LN2020H	*Rhinolophus ferrumequinum*	OK017800	09/2020	Liaoning
43	Bat-SARSr-CoV-ZXC21	*Rhinolophus pusillus*	MG772934	07/2015	Zhejiang
44	Bat-SARSr-CoV-ZC45	*Rhinolophus pusillus*	MG772933	02/2017	Zhejiang
45	Bat-SARSr-CoV-HKU3-1	*Rhinolophus sinicus*	DQ022305.2	17/02/2005	Hong Kong
46	Bat-SARSr-CoV-HKU3-2	*Rhinolophus sinicus*	DQ084199	24/02/2005	Hong Kong
47	Bat-SARSr-CoV-HKU3-3	*Rhinolophus sinicus*	DQ084200	17/03/2005	Hong Kong
48	Bat-SARSr-CoV-HKU3-4	*Rhinolophus sinicus*	GQ153539	20/07/2005	Hong Kong
49	Bat-SARSr-CoV-HKU3-5	*Rhinolophus sinicus*	GQ153540	20/09/2005	Hong Kong
50	Bat-SARSr-CoV-HKU3-6	*Rhinolophus sinicus*	GQ153541	16/12/2005	Hong Kong
51	Bat-SARSr-CoV-HKU3-7	*Rhinolophus sinicus*	GQ153542	15/02/2006	Guangdong
52	Bat-SARSr-CoV-HKU3-8	*Rhinolophus sinicus*	GQ153543	15/02/2006	Guangdong
53	Bat-SARSr-CoV-HKU3-9	*Rhinolophus sinicus*	GQ153544	28/08/2006	Hong Kong
54	Bat-SARSr-CoV-HKU3-10	*Rhinolophus sinicus*	GQ153545	28/08/2006	Hong Kong
55	Bat-SARSr-CoV-HKU3-11	*Rhinolophus sinicus*	GQ153546	07/03/2007	Hong Kong
56	Bat-SARSr-CoV-HKU3-12	*Rhinolophus sinicus*	GQ153547	15/05/2007	Hong Kong
57	Bat-SARSr-CoV-HKU3-13	*Rhinolophus sinicus*	GQ153548	15/11/2007	Hong Kong
58	Bat-SARSr-CoV-JX2021P	*Rhinolophus sinicus*	OK017845	07/2021	Jiangxi
59	Bat-SARSr-CoV-JX2021AC	*Rhinolophus sinicus*	OK017836	07/2021	Jiangxi
60	Bat-SARSr-CoV-JX2021C	*Rhinolophus sinicus*	OK017837	07/2021	Jiangxi
61	Bat-SARSr-CoV-JX2021G	*Rhinolophus sinicus*	OK017838	07/2021	Jiangxi
62	Bat-SARSr-CoV-JX2021J	*Rhinolophus sinicus*	OK017839	07/2021	Jiangxi
63	Bat-SARSr-CoV-JX2021K	*Rhinolophus sinicus*	OK017840	07/2021	Jiangxi
64	Bat-SARSr-CoV-JX2021L	*Rhinolophus sinicus*	OK017841	07/2021	Jiangxi
65	Bat-SARSr-CoV-JX2021M	*Rhinolophus sinicus*	OK017842	07/2021	Jiangxi
66	Bat-SARSr-CoV-JX2021N	*Rhinolophus sinicus*	OK017843	07/2021	Jiangxi
67	Bat-SARSr-CoV-JX2021O	*Rhinolophus sinicus*	OK017844	07/2021	Jiangxi

68	Bat-SARSr-CoV-HN2021A	*Rhinolophus pusillus*	OK017803	02/2021	Hunan
69	Bat-SARSr-CoV-HN2021B	*Rhinolophus pusillus*	OK017804	02/2021	Hunan
70	Bat-SARSr-CoV-HN2021G	*Rhinolophus pusillus*	OK017805	03/2021	Hunan
71	Bat-SARSr-CoV-HN2021C	*Rhinolophus sinicus*	OK017832	02/2021	Hunan
72	Bat-SARSr-CoV-HN2021D	*Rhinolophus sinicus*	OK017833	02/2021	Hunan
73	Bat-SARSr-CoV-HN2021E	*Rhinolophus sinicus*	OK017834	03/2021	Hunan
74	Bat-SARSr-CoV-HN2021F	*Rhinolophus sinicus*	OK017835	03/2021	Hunan
75	Bat-SARSr-CoV-GD2019A	*Rhinolophus sinicus*	OK017825	08/2019	Guangdong
76	Bat-SARSr-CoV-GD2019B	*Rhinolophus sinicus*	OK017826	08/2019	Guangdong
77	Bat-SARSr-CoV-GD2019D	*Rhinolophus sinicus*	OK017827	09/2019	Guangdong
78	Bat-SARSr-CoV-GD2019E	*Rhinolophus sinicus*	OK017828	09/2019	Guangdong
79	Bat-SARSr-CoV-GD2017F	*Rhinolophus affinis*	OK017792	08/2017	Guangdong
80	Bat-SARSr-CoV-GD2017G	*Rhinolophus sinicus*	OK017813	09/2017	Guangdong
81	Bat-SARSr-CoV-GD2017H	*Rhinolophus sinicus*	OK017814	09/2017	Guangdong
82	Bat-SARSr-CoV-GD2017I	*Rhinolophus sinicus*	OK017815	09/2017	Guangdong
83	Bat-SARSr-CoV-GD2017J	*Rhinolophus sinicus*	OK017816	09/2017	Guangdong
84	Bat-SARSr-CoV-GD2017K	*Rhinolophus sinicus*	OK017817	09/2017	Guangdong
85	Bat-SARSr-CoV-GD2017L	*Rhinolophus sinicus*	OK017818	09/2017	Guangdong
86	Bat-SARSr-CoV-GD2017M	*Rhinolophus sinicus*	OK017819	09/2017	Guangdong
87	Bat-SARSr-CoV-GD2017N	*Rhinolophus sinicus*	OK017820	09/2017	Guangdong
88	Bat-SARSr-CoV-GD2017O	*Rhinolophus sinicus*	OK017821	09/2017	Guangdong
89	Bat-SARSr-CoV-GD2017P	*Rhinolophus sinicus*	OK017822	09/2017	Guangdong
90	Bat-SARSr-CoV-GD2017Q	*Rhinolophus sinicus*	OK017823	09/2017	Guangdong
91	Bat-SARSr-CoV-GD2017W	*Rhinolophus sinicus*	OK017824	09/2017	Guangdong
92	Bat-SARSr-CoV-GD2016B	*Rhinolophus sinicus*	OK017812	07/2016	Guangdong
93	Bat-SARSr-CoV-FJ2021M	*Rhinolophus sinicus*	OK017811	05/2021	Fujian
94	Bat-SARSr-CoV-FJ2021A	*Rhinolophus sinicus*	OK017808	05/2021	Fujian
95	Bat-SARSr-CoV-FJ2022D	*Rhinolophus sinicus*	OK017809	05/2021	Fujian
96	Bat-SARSr-CoV-FJ2021E	*Rhinolophus sinicus*	OK017810	05/2021	Fujian
97	Bat-SARSr-CoV-BtKY72	*Rhinolophus* sp.	KY352407	08/2007	Kenya
