# Supplementary material for: Epidemiology and Genomic Characterization of Two Novel SARS-Related Coronaviruses in Horseshoe Bats from Guangdong, China
Source: mBio. 2022 Apr 25;13(3):e00463-22. doi: 10.1128/mbio.00463-22 (PMC9239062; doi:10.1128/mbio.00463-22)
Supplement: FIG S5 [file mbio.00463-22-sf005.pdf]

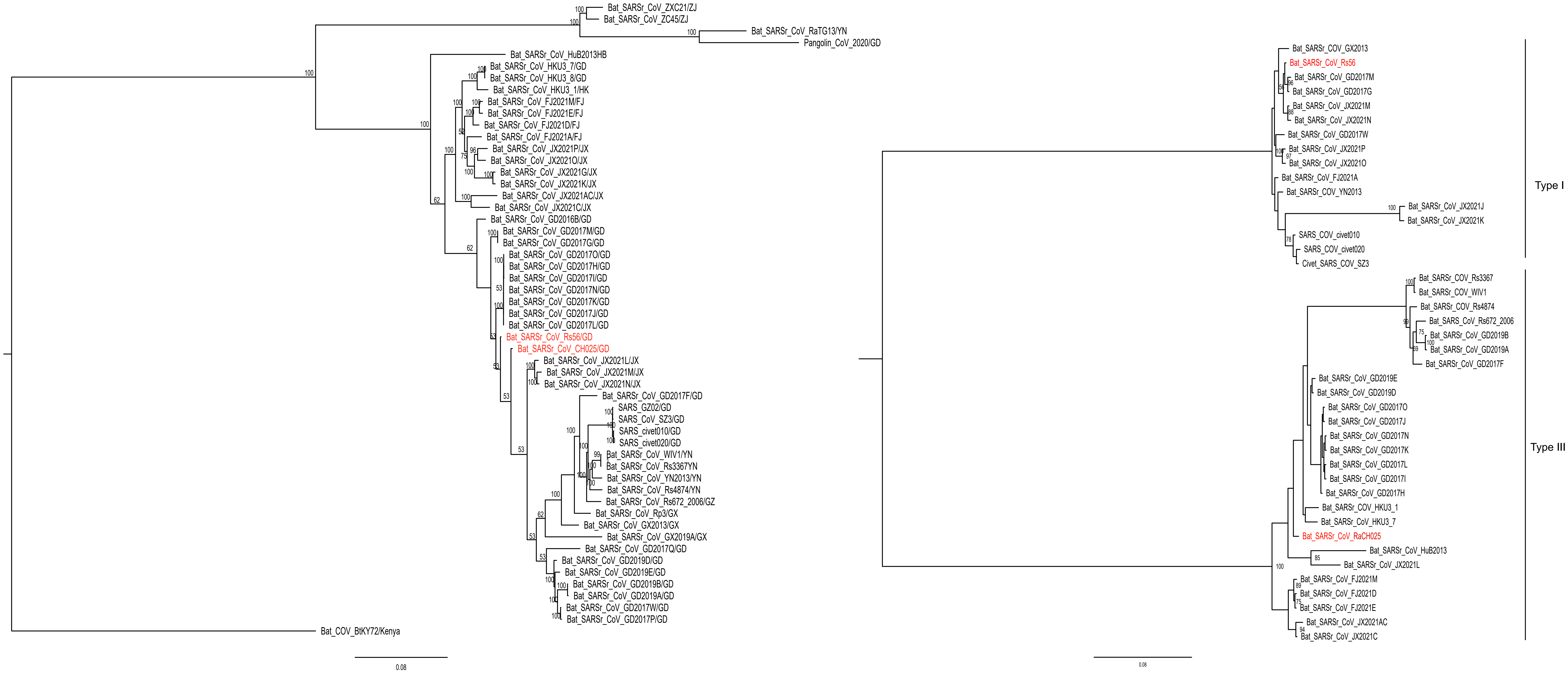

Fig. S5 Phylogenetic trees based on nucleotide sequences of ORF1ab (A) and ORF8 (B). The trees were constructed by the maximum likelihood method using MrBayes approach employing the GTR+I+G (A) and GTR+G (B) nucleotide substitution model. The red letters represent the SARSr-CoV strains isolated from bats from Guangdong in this study.
