# Supplementary material for: Epidemiology and Genomic Characterization of Two Novel SARS-Related Coronaviruses in Horseshoe Bats from Guangdong, China
Source: mBio. 2022 Apr 25;13(3):e00463-22. doi: 10.1128/mbio.00463-22 (PMC9239062; doi:10.1128/mbio.00463-22)
Supplement: FIG S6 [file mbio.00463-22-sf006.pdf]

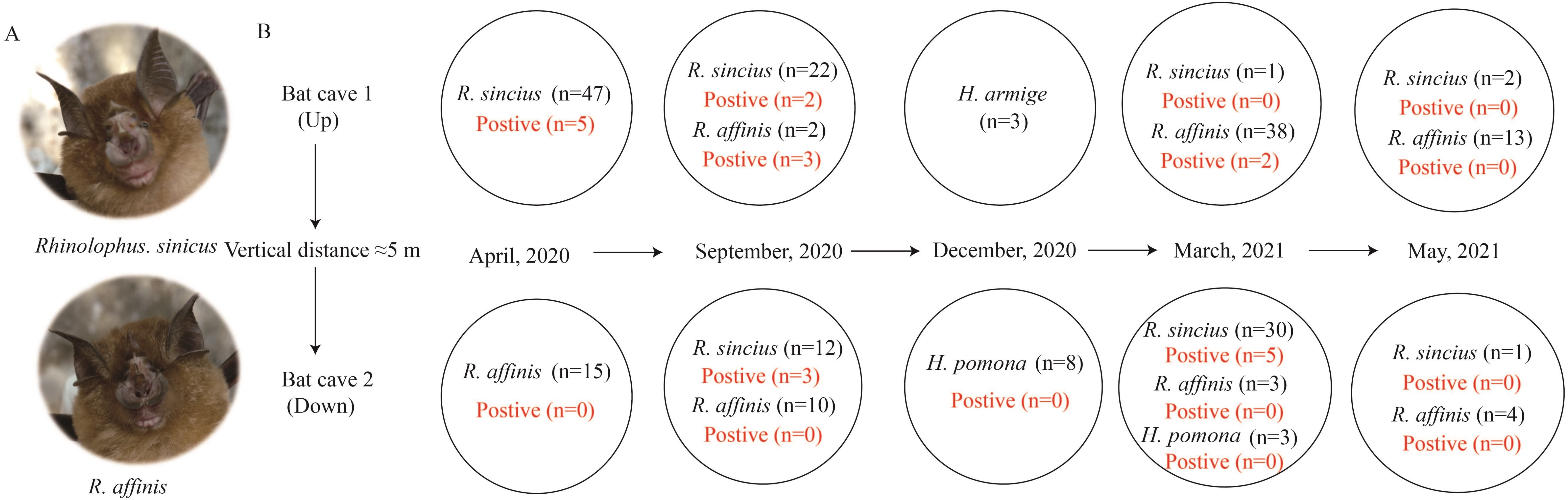

Fig.S6 Bat's form and schematic diagram of bat roosting cave. (A) ***Rhinolophus sinicus*** and ***R. affinis***. (B) Schematic diagram of bat roosting cave.
